# TFEB controls expression of human syncytins during cell–cell fusion

**DOI:** 10.1101/gad.351633.124

**Published:** 2024-08-01

**Authors:** Meagan N. Esbin, Liza Dahal, Vinson B. Fan, Joey McKenna, Eric Yin, Xavier Darzacq, Robert Tjian

**Affiliations:** 1 Department of Molecular and Cell Biology, University of California Berkeley, Berkeley, California 94720, USA;; 2 Howard Hughes Medical Institute, University of California, Berkeley, California 94720, USA

**Keywords:** TFEB, cell fusion, placenta, single-molecule imaging, syncytin, transcription factor

## Abstract

During human development, a temporary organ is formed, the placenta, which invades the uterine wall to support nutrient, oxygen, and waste exchange between the mother and fetus until birth. Most of the human placenta is formed by a syncytial villous structure lined by syncytialized trophoblasts, a specialized cell type that forms via cell–cell fusion of underlying progenitor cells. Genetic and functional studies have characterized the membrane protein fusogens Syncytin-1 and Syncytin-2, both of which are necessary and sufficient for human trophoblast cell–cell fusion. However, identification and characterization of upstream transcriptional regulators regulating their expression have been limited. Here, using CRISPR knockout in an in vitro cellular model of syncytiotrophoblast development (BeWo cells), we found that the transcription factor TFEB, mainly known as a regulator of autophagy and lysosomal biogenesis, is required for cell–cell fusion of syncytiotrophoblasts. TFEB translocates to the nucleus, exhibits increased chromatin interactions, and directly binds the Syncytin-1 and Syncytin-2 promoters to control their expression during differentiation. Although TFEB appears to play a critical role in syncytiotrophoblast differentiation, ablation of TFEB largely does not affect lysosomal gene expression or lysosomal biogenesis in differentiating BeWo cells, suggesting a previously uncharacterized role for TFEB in controlling the expression of human syncytins.

## Abstract (precis)

In this study, Esbin et al. report that the transcription factor TFEB controls the expression of placental fusogens Syncytin-1 and Syncytin-2, driving the cell–cell fusion of syncytiotrophoblasts. This work reveals the critical new role for TFEB in controlling the expression of human syncytins and placental development that extends beyond its known roles in lysosomal biogenesis.

Shortly after implantation of a fertilized blastocyst, the placenta, a temporary organ, begins to develop alongside the human fetus, which provides vital sustenance and exchange during pregnancy and sets the fetus up for a healthy life after birth (Hubrecht 1889; Burton and Fowden 2015; Turbeville and Sasser 2020). Many of the underlying molecular mechanisms driving development of the placenta remain enigmatic yet vital to understand. The placenta is thought to be the basis of many obstetrical complications, so its dual role in maintaining both fetal and maternal health during pregnancy suggests that a deeper understanding of human placental development provides an opportunity to both support healthy neonate development and combat the rising maternal mortality rates plaguing the U.S. and the world (Brosens et al. 2011; Ghulmiyyah and Sibai 2012; https://stacks.cdc.gov/view/cdc/124678). During early development of the human placenta, trophoblast stem cells generate three main lineages, where cytotrophoblast progenitor cells (CTBs; mononuclear stem cells that proliferate and support placental growth) differentiate into two main functional cell types: syncytiotrophoblasts (STBs; multinucleated syncytia formed via cell–cell fusion of cytotrophoblasts that form the outermost barrier between the fetal and maternal circulation) and extravillous trophoblasts (EVTs; a mononuclear specialized subset of cytotrophoblasts that invade and remodel the maternal spiral arteries to modulate blood flow into the placenta) (Turco and Moffett 2019). The regulation of syncytialization within the placental villous appears to be vital for placental and maternal health. Aberrant syncytialization has been observed concomitant with several important obstetrical conditions, including preeclampsia (reduced syncytialization) and fetal growth restriction (excess syncytialization) (Vargas et al. 2011; Mukherjee et al. 2021; Shao et al. 2021).

Differentiation of syncytiotrophoblasts is characterized by cell–cell fusion, secretion of pregnancy hormones including hCG, nuclear enlargement, and exit from mitosis while differentiated cells remain transcriptionally active (Galton 1962; Pierce and Midgley 1963; Ellery et al. 2009). The molecular mediators of cell–cell fusion in human syncytiotrophoblasts are evolutionarily modern endogenous retroviral envelope proteins, Syncytin-1 and Syncytin-2, which are membrane proteins with high expression in the cytotrophoblasts and syncytiotrophoblasts of the human placenta (Blond et al. 2000; Mi et al. 2000; Blaise et al. 2003; Roberts et al. 2021). Syncytin-1 and Syncytin-2 have fusogenic activity and are sufficient to induce cell–cell fusion in cells expressing their receptors, SLC1A4/5 and MFSD2A, respectively (Blond et al. 2000; Esnault et al. 2008; Toufaily et al. 2013). Decades of research have illustrated the important role of many cellular factors in orchestrating the differentiation and cell–cell fusion of syncytiotrophoblasts, but only one direct upstream regulator of placental syncytialization has been identified: the transcription factor GCM1 (Papuchova and Latos 2022). Interestingly, GCM1 has been co-opted evolutionarily to be capable of regulating the sequence-unrelated syncytin genes in both humans (Yu et al. 2002; Lin et al. 2005; Liang et al. 2010) and mice (Anson-Cartwright et al. 2000; Schreiber et al. 2000; Simmons et al. 2008; Bainbridge et al. 2012), and GCM1 has been shown to directly bind the promoters of Syncytin-1, Syncytin-2, and MFSD2A (Baczyk et al. 2009; Liang et al. 2010; Jeyarajah et al. 2022). To date, other direct regulators of Syncytin-1 or Syncytin-2 expression have not been identified.

The transcription factor TFEB is a potentially interesting, conserved candidate for regulating placental differentiation. TFEB is highly expressed in syncytiotrophoblasts and cytotrophoblasts within the human placenta (Vento-Tormo et al. 2018). Like GCM1, homozygous knockout of the transcription factor *TFEB* is lethal during gestation (d9.5) due to failure to form the syncytialized labyrinth layer of the murine placenta (Steingrímsson et al. 1998; Anson-Cartwright et al. 2000). TFEB belongs to a small family (along with TFE3, MITF, and TFEC) of basic helix–loop–helix DNA binding transcription factors that homodimerize and heterodimerize to bind a subset of E-box motifs in the genome (Fisher et al. 1991; Tan et al. 2022). Within the nucleus of human cells, TFEB is thought to be a master regulator of lysosomal biogenesis by transactivating the coordinated lysosomal expression and regulation (CLEAR) gene network (Sardiello et al. 2009; Palmieri et al. 2011). TFEB's cytoplasmic-to-nuclear import is negatively controlled largely via phosphorylation of TFEB by mTOR and other kinases (Roczniak-Ferguson et al. 2012; Settembre et al. 2012; Puertollano et al. 2018).

In patients with the placental disease preeclampsia, protein levels of TFEB are reduced in placental tissue compared with age-matched controls (Nakashima et al. 2020a,b). Consistent with its effects on TFEB nuclear localization, perturbation of mTOR signaling has also been shown to affect trophoblast syncytialization: In BeWo cells, treatment with the mTOR inhibitor Rapamycin increases cell fusion and hCG production, whereas concomitant treatment with the mTOR activator MHY1485 erases these effects (Furuta et al. 2022). In contrast, for cases of fetal growth restriction (FGR), researchers have found reduced mTOR phosphorylation and excess cell–cell fusion (Shao et al. 2021). Although these findings may point to TFEB as an interesting potential candidate in syncytiotrophoblast differentiation, to date, TFEB's direct role in human placental development has not been investigated.

## Results

**Figure 1.**
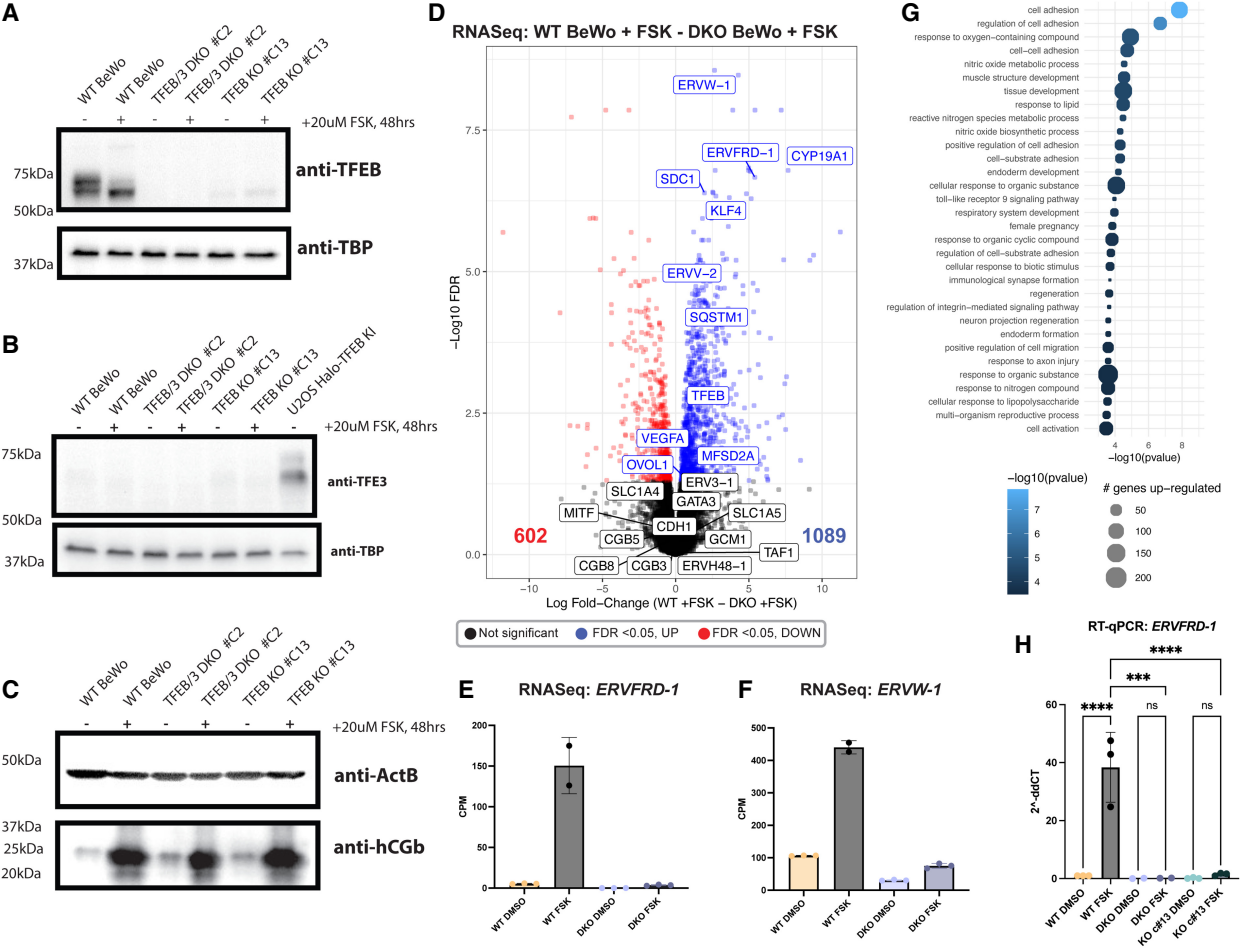
Perturbation of TFEB and TFE3 in BeWo cells using CRISPR KO. (*A*–*C*) Western blotting of TFEB (*A*), TFE3 (*B*), and hCG (*C*) expression in wild-type and CRISPR KO BeWo cells. (*D*) Volcano plot of RNA-seq data comparing Forskolin-treated wild-type BeWo cells with Forskolin-treated TFEB/TFE3 DKO BeWo cells. Genes that are significantly higher in the Forskolin-treated DKO cells are shown in red, and genes that are significantly lower in the Forskolin-treated DKO cells are shown in blue. (*E*,*F*) Normalized CPM values from the RNA-seq data showing expression of *ERVFRD-1* (Syncytin-2) (*E*) or *ERVW-1* (*F*) across samples. Data points indicate separate RNA-seq replicates, and error bars show standard deviation. (*G*) GO analysis of genes upregulated in Forskolin-treated WT BeWo compared with Forskolin-treated DKO BeWo cells. The top biological process GO terms are shown. (*H*) Expression of *ERVFRD-1* transcripts measured by RT-qPCR and quantified by the ΔΔ*Ct* method. Statistical significance from an ordinary one-way ANOVA with Tukey's multiple comparisons test is shown. (ns) Not significant, (***) *P* < 0.001, (****) *P* < 0.0001.

To assess TFEB's roles in human syncytiotrophoblast differentiation, we used CRISPR/Cas9 to genetically delete *TFEB* in BeWo cells (an in vitro model of syncytiotrophoblast differentiation), which inducibly differentiate and fuse upon treatment with the cAMP activator Forskolin (Pattillo and Gey 1968; Wice et al. 1990; Orendi et al. 2010). To prevent genetic compensation from TFE3 as seen in other cellular systems (Pastore et al. 2016; Annunziata et al. 2019; Tan et al. 2021), we started by creating a homozygous *TFEB* and *TFE3* double-knockout BeWo cell line by transfecting plasmids encoding Cas9-Venus and 10 sgRNAs targeting the N-terminal and C-terminal regions of *TFEB* and *TFE3* to induce genetic deletions within the two protein-coding loci (see Table 1 in the Materials and Methods; Supplemental Fig. S1A). One clone (#C2) was confirmed to have deletions in the coding regions of *TFEB* and *TFE3* and complete loss of TFEB and TFE3 protein expression by Western blotting (Fig. 1A,B). Although TFEB was moderately expressed in wild-type BeWo cells, TFE3 was virtually undetectable by Western blotting due to its low expression (Fig. 1B; Supplemental Fig. S1B). Because TFE3 was not appreciably expressed in wild-type BeWo cells, we additionally created *TFEB*-only KO BeWo cells by nucleofecting BeWo cells with Cas9 RNPs containing a single sgRNA targeted to induce indels at the N terminus of *TFEB*. The edit yielded one clone (TFEB KO #c13) with significant depletion of TFEB via induced indels (Supplemental Fig. S1C). By Western blotting, this clone had ∼11% remaining TFEB expression (Fig. 1A). Notably, the TFEB KO BeWo cells, like the wild type, do not show any appreciable TFE3 expression, and compensation at the RNA level was not observed, indicating that TFEB's loss is not compensated for by upregulation of TFE3 in BeWo cells (Fig. 1B). In both clones, some characteristics of differentiation appear entirely unperturbed upon TFEB loss, including the ability to produce hCG and the enlargement of nuclei upon differentiation with Forskolin (Fig. 1C; Supplemental Fig. S1D).

**Table 1.** Guide RNAs for TFEB/TFE3 KO

Target	Number	Guide RNA sequence (5′–3′)
TFEB Cterm	67F	TCTGTCCACCATGTCCCCCG
TFEB Cterm	8R	GCGGTAGCAGTGAGTCGTCC
TFEB Cterm	132F	AGGGCGATGTGCTGTGACCC
TFEB Nterm	121R	CAACCCTATGCGTGACGCCA
TFEB Nterm	138F	CCACCATGGCGTCACGCATA
TFE3 Cterm	173R	AGGCGGGGCCTCATCCTGAC
TFE3 Cterm	22R	ATCGGAGGCAGCCCGCAGTG
TFE3 Nterm	139R	TACGCCATCCCGAGCTGGTT
TFE3 Nterm	163F	ACCAGCTCGGGATGGCGTAG
TFE3 Nterm	150F	CTCATGCGGCCGAACCAGCT

To identify genes that require TFEB for expression during BeWo differentiation, we performed RNA-seq in wild-type and DKO #C2 BeWo cells treated for 48 h with 0.1% DMSO or 20 µM Forskolin to induce differentiation (Roczniak-Ferguson et al. 2012; Settembre et al. 2012). Interestingly, differential gene expression analysis identified >1000 genes whose expression was significantly higher in Forskolin-treated wild-type cells compared with the Forskolin-treated DKO BeWo cells (Fig. 1D). Among those genes most significantly dysregulated were known markers of syncytiotrophoblast differentiation, including the syncytin loci *ERVFRD-1* (Syncytin-2) and *ERVW-1* (Syncytin-1) as well as syncytiotrophoblast-expressed genes *ERVV-2*, *SDC-1, KFL4*, and *CYP19A1* (aromatase) (Fig. 1E,F; Hudon Thibeault et al. 2018; Roberts et al. 2021). RT-qPCR confirmed significant defects in upregulating *ERVFRD-1* during differentiation in TFEB/3 DKO and TFEB KO #c13 cells (Fig. 1H). Notably, *GCM1* transcripts were not significantly affected in the RNA-seq, suggesting that TFEB's role is not directly upstream of *GCM1* mRNA expression (Fig. 1D). Transcription of the hCG hormone cluster genes expressed in BeWo cells (*CB3*, *CGB5*, and *CB8*) was also not affected by TFEB loss, congruent with the unchanged protein expression measured by Western blotting (Fig. 1C,D). GO classification of these ∼1000 genes was significantly enriched in categories relevant to cell–cell fusion, including cell adhesion, cell substrate adhesion, integrin signaling, and female pregnancy, and cellular compartment analysis indicated enrichment in extracellular matrix and cell membrane processes (Fig. 1G; Supplemental Fig. S2A). To identify genes that may be responsive to nuclear TFEB, we also performed RNA-seq in wild-type and DKO #C2 BeWo cells treated for 2 or 48 h with 250 nM Torin1, an mTOR inhibitor shown to induce nuclear TFEB localization (Roczniak-Ferguson et al. 2012; Settembre et al. 2012). Surprisingly, treatment of cells with Torin1 for 48 h elevated levels of STB marker genes including *ERVFRD-1*, *ERVW-1*, *SDC-1*, *OVOL1*, and *CYP19A1* to levels similar to those observed with Forskolin treatment (Supplemental Fig. S2C). Even after just 2 h of treatment with Torin1, *ERVFRD-1* was identified as one of the first genes to be significantly upregulated by acute Torin1 treatment (Supplemental Fig. S2B), suggesting a link between nuclear TFEB import and activation of syncytins in BeWo cells.

**Figure 2.**
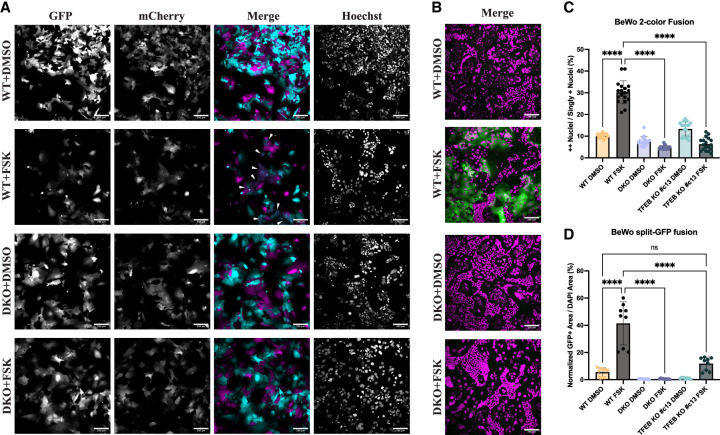
KO of TFEB/TFE3 in BeWo cells causes a functional defect in cell–cell fusion. (*A*) Two-color cell–cell fusion experiments coculturing two populations of mCherry- and GFP-expressing BeWo cells were imaged on a spinning-disk confocal microscope. Individual channels are shown in grayscale. In the merged composite image, the GFP channel is represented in cyan, and the mCherry channel is represented in magenta. White arrowheads indicate fused syncytial areas in the Forskolin-treated wild-type cells. Scale bar, 200 μm. (*B*) Split-GFP cell–cell fusion experiments coculturing GFP1–10-expressing BeWo cells with GFP11-expressing 293T cells and acquiring images on a spinning-disk confocal microscope. In the merged composite image, the GFP channel is represented in green, and the Hoechst channel is represented in magenta. Fused syncytial areas are shown by the reconstitution of GFP fluorescence, shown in green. Scale bar, 200 μm. (*C*) Quantification of the two-color cell–cell fusion experiments shown in *A*. (*D*) Quantification of the split-GFP cell–cell fusion experiments shown in *B*. For *C* and *D*, statistical significance from an ordinary one-way ANOVA with Tukey's multiple comparisons test is shown. (ns) Not significant, (****) *P* < 0.0001.

Given the apparent role for TFEB in syncytin expression, we next sought to determine whether the loss in syncytin transcripts results in a functional defect in cell–cell fusion. Two quantitative imaging-based assays were used to assess cell–cell fusion of wild-type, DKO, and TFEB KO BeWo cells upon 48 h Forskolin-induced fusion. First, a two-color BeWo:BeWo fusion assay was performed by coculturing BeWo cell lines transduced with lentivirus encoding for either mCherry or GFP (Fig. 2A; Supplemental Fig. S3A). Upon imaging of Hoechst-labeled nuclei in these two-color fusion experiments, quantification of mCh/GFP double-positive nuclei indicated the fused population (Supplemental Fig. S3C). Forskolin treatment significantly increased the proportion of double-positive over singly positive nuclei in the wild-type cells, whereas this effect was nullified in the DKO and TFEB KO cells (Fig. 2C). Additionally, re-expression of TFEB in the BeWo DKO cells restored cell–cell fusion and phenotypes of differentiating syncytiotrophoblasts, including clustered, enlarged nuclei (Supplemental Fig. S4). In our RNA-seq data sets, expression of the Syncytin-2 receptor *MFSD2A* was also lower in Forskolin-treated TFEB/3 DKO cells compared with wild-type cells, whereas the Syncytin-1 receptors *SLC1A4/5* were unaffected (Fig. 1D). Because syncytins are unidirectional fusogens (i.e., they only need to be present on one membrane to induce fusion), loss of cell–cell fusion in a BeWo:BeWo fusion assay could be due to either loss of the fusogens themselves (Syncytin-1 or Syncytin-2) on one membrane or loss of their receptors (SLC1A5 or MFSD2A) on the opposing membrane, respectively (Mi et al. 2000). To assess whether the TFEB-associated loss in syncytin expression alone significantly impacted cell–cell fusion, we also assessed cell–cell fusion between the wild-type, DKO, and TFEB KO BeWo cells with 293T cells. As observed by Mi et al. (2000), a donor cell line that constitutively expresses the necessary receptor proteins at high level (in their case, MCF7; in our case, 293T) can be seen to fuse and flatten into BeWo cells in a fully syncytin-dependent manner. In our experiment, we used a split-GFP approach where the respective BeWo cell lines were transduced with a lentivirus to express GFP1–10 and cocultured with 293T cells expressing GFP11. In wild-type cells, upon treatment with Forskolin, we observed large sheets of fused GFP-positive regions, indicating cell–cell fusion between the 293T and BeWo cells, whereas in Forskolin-treated DKO and TFEB KO cells, the GFP-positive area was dramatically reduced (Fig. 2B,D; Supplemental Fig. S3B). Notably, there was a severely reduced capability of the TFEB KO #c13 cells to fuse with the 293T cells (Fig. 2D; Supplemental Fig. S3B). We did observe a very small residual capacity of the TFEB KO #c13 cells to fuse with the 293T cells and attribute this to the small amount of remaining TFEB protein present in these cells (11%) that led to a small but detectable increase in Syncytin-2 (*ERVFRD-1*) transcript levels upon Forskolin treatment (Fig. 1H). In sum, TFEB loss results in a severe defect in the normal upregulation of syncytins during differentiation that results in significant defects in cell–cell fusion.

**Figure 3.**
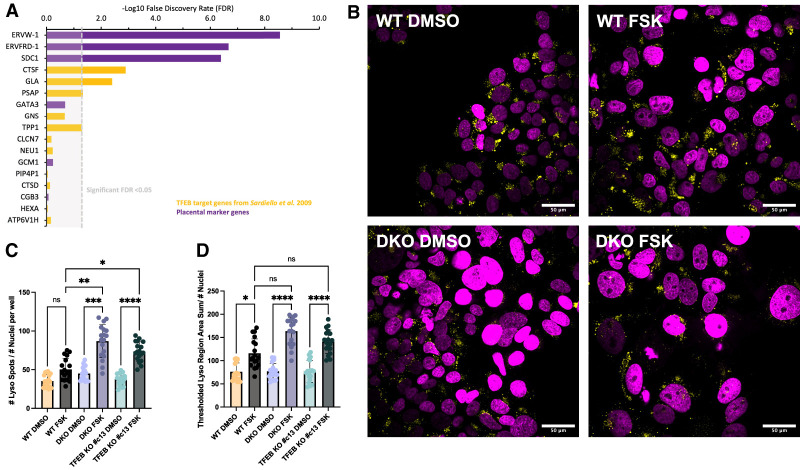
TFEB/3 KO in BeWo cells has minimal effects on lysosomal biogenesis or lysosomal gene expression. (*A*) Differential gene expression analysis of previously identified lysosomal TFEB target genes (yellow bars) and selected syncytiotrophoblast marker genes (purple bars). The −log_10_ false discovery rate (FDR) of the comparison between Forskolin-treated wild-type BeWo cells and Forskolin-treated TFEB/3 DKO BeWo cells is shown with a significance cutoff of FDR = 0.05, equivalent to −log_10_(FDR) = 1.30. (*B*) Live lysosomes were imaged in DMSO-treated or Forskolin-treated BeWo cells by staining with Lysoview-540 and imaging on a spinning-disk confocal microscope. In the merged composite image, the Hoechst channel is represented in magenta, and the Lysoview staining is shown in yellow. Scale bar, 50 μm. (*C*) The normalized number of lysosomes was quantified by the per-well mean of total lysosomal spots detected per image divided by the total number of nuclei per image. (*D*) The normalized total lysosomal area is quantified by the per-well mean of 540 nm-positive thresholded region summed area per image divided by the total number of nuclei per image. For *C* and *D*, statistical significance from Kruskal–Wallis ANOVA test with Dunn's multiple comparison test is shown. (ns) Not significant, (*) *P* < 0.05, (**) *P* < 0.01, (***) *P* < 0.001, (****) *P* < 0.0001.

TFEB's main transcriptional role, as previously characterized in HeLa cells, is to control expression of many lysosomal and autophagy genes enriched in 10 bp expanded E-box (CLEAR) motifs, termed the CLEAR gene network (Sardiello et al. 2009; Palmieri et al. 2011). Expression analysis of 11 CLEAR network TFEB target genes identified by Sardiello et al. (2009) in our BeWo RNA-seq data set yielded only three that were significantly different between the Forskolin-treated wild-type and DKO cells, whereas most remained unchanged (Fig. 3A). Even though only three of the lysosomal genes were perturbed, in addition to gene expression data, we next sought to investigate whether TFEB loss had any effect phenotypically on lysosomal organelle biogenesis. We stained BeWo cells with the live-cell pH-sensitive lysosomal marker Lysoview-540 (Biotium) and imaged live lysosomes in DMSO and Forskolin-treated BeWo cells (Fig. 3B). The number of lysosomes and the total area occupied by lysosomes were similar in the wild-type cells compared with the DKO and TFEB KO BeWo cells (Fig. 3C,D; Supplemental Fig. S3D). Following from our findings that TFEB loss in BeWo cells does not majorly perturb lysosomal gene expression, we found that lysosomal biogenesis during Forskolin treatment proceeds regardless of TFEB knockout and, indeed, with even larger gains in lysosomes in the differentiating TFEB DKO and TFEB KO #c13 cells than in wild-type cells (Fig. 3C,D). Thus, lysosomal biogenesis in BeWo cells is activated upon Forskolin differentiation but seems to be largely independent from TFEB expression, suggesting that an alternative or redundant factor may instead be controlling lysosomal gene expression in placental cells.

**Figure 4.**
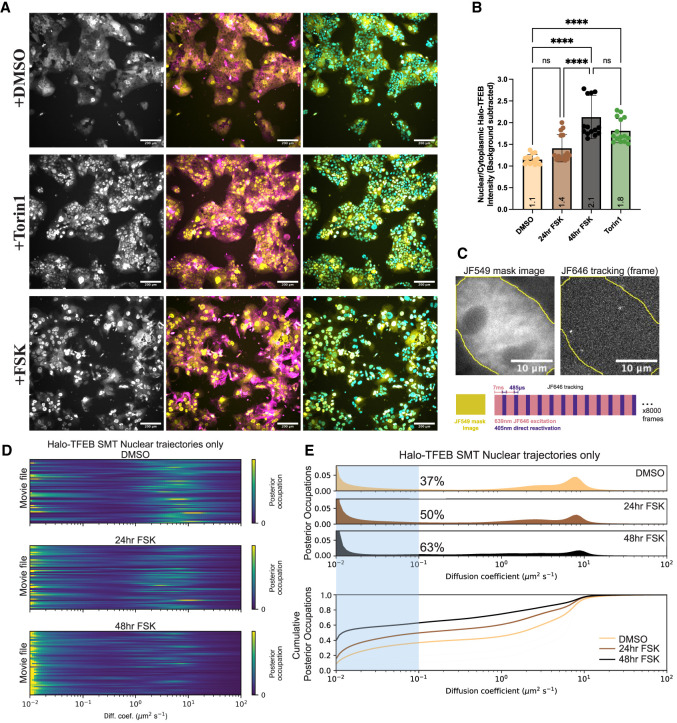
TFEB translocates into the nucleus and binds chromatin during syncytiotrophoblast differentiation. (*A*) Live-cell spinning-disk confocal imaging of Halo-TFEB after treatment with Torin1 for 2 h or treatment with Forskolin for 48 h. The Halo-TFEB channel alone is shown in grayscale (*left*), followed by a composite image showing Halo-TFEB (yellow) with the membrane stain Biotium MembraneSteady-488 nm (magenta) (*middle*), and Halo-TFEB (yellow) with Hoechst-stained nuclei (cyan). Scale bar, 200 μm. (*B*) Quantification of the nuclear/cytoplasmic ratio of Halo-TFEB in *A*. Statistical significance from an ordinary one-way ANOVA with Tukey's multiple comparisons test is shown. (ns) Not significant, (****) *P* < 0.0001. (*C*, *top*) Diagram of single-molecule tracking of Halo-TFEB performed in BeWo cells. An example JF549 mask image (*left*) and a single frame from a JF646-tracking movie (*right*) are shown, with the binarized mask outline used for segmenting nuclear versus external trajectories shown overlaid in yellow. Scale bar, 10 μm. The *bottom* diagram shows how image acquisition was performed by acquiring a single JF549 image used for masking followed by stroboscopic illumination and activation of dark JF646 molecules using 405 nm activation in the camera readout time. (*D*) Heat maps of diffusion coefficients following Bayesian analysis of single-molecule trajectory data of Halo-TFEB in BeWo cells in different drug conditions. Each row corresponds to the distribution of posterior occupations for a single movie file. (*E*) The distribution of diffusion coefficient occupancies for nuclear segmented trajectories of Halo-TFEB in different drug conditions. The fraction bound (calculated by the fraction of the distribution with a diffusion coefficient of <0.1 μm^2^/sec) is shown highlighted with the blue region and the quantified values displayed as black percentages. The cumulative distribution function (CDF) of this same distribution is shown *below*.

To more closely examine TFEB's mechanism in controlling syncytiotrophoblast gene expression, we investigated TFEB's nuclear and cytoplasmic partitioning during Forskolin treatment. We performed high-throughput confocal imaging of JF646-stained BeWo cells overexpressing Halo-3xFLAG-TFEB driven by an L30 promoter and treated with DMSO or Forskolin. In untreated cells, TFEB localization is variable, with most cells exhibiting cytoplasmic TFEB and some showing predominantly nuclear TFEB (Fig. 4A). Segmentation and quantification of the nuclear/cytoplasmic ratio of TFEB demonstrated an approximately twofold increase (from ∼1 to ∼2) in nuclear/cytoplasmic TFEB after 48 h treatment with Forskolin, similar to the effect of acute 1 h Torin1 treatment (Fig. 4B). Western blot staining of TFEB in BeWo cells also showed a downward shift in the apparent molecular weight of the anti-TFEB band upon Forskolin treatment (Fig. 1A), a common signature of TFEB being dephosphorylated, an observation consistent with current evidence that TFEB's shuttling to the nucleus is concomitant with its dephosphorylation (Martina et al. 2012; Roczniak-Ferguson et al. 2012; Settembre et al. 2012).

Given TFEB's redistribution to the nucleus upon Forskolin treatment, we investigated whether TFEB's chromatin binding within the nucleus was altered during differentiation. To sensitively measure TFEB–chromatin binding in live cells, we performed single-molecule tracking (SMT) of BeWo cells stably overexpressing Halo-TFEB. SMT allowed us to assess the proportion of TFEB molecules engaged in chromatin binding (“bound”) versus freely diffusing (“free”) by collecting trajectories from ∼60,000 individual TFEB molecules across ∼60 cells per condition. We labeled Halo-TFEB-overexpressing BeWo cells with two JF dyes in tandem: sparse (25 nM) JFX646 for tracking and more dense (50 nM) JF549 to assess the overall distribution of TFEB in the nucleus and cytoplasm. We then performed stroboscopic fast tracking (7 msec frame rate) with interleaved direct photoreactivation of dark molecules using 405 nm light to accurately capture fast-moving molecules (Fig. 4C). Single-molecule tracking of control constructs Halo-H2B (a highly chromatin-bound protein) and Halo-NLS (a diffusing control) yielded bound fractions of ∼85% and ∼9%, respectively, indicating the dynamic range of such an assay to detect immobile versus freely diffusing biological molecules (Supplemental Fig. S5A,B; Supplemental Movies S1, S2). Additionally, Forskolin treatment alone did not induce a major change in the diffusion of the Halo-NLS or Halo-H2B controls (Supplemental Fig. S5A,B). In DMSO-treated cells, Halo-TFEB diffusion modeled using saSPT exhibited a diffusion spectrum with approximately three modes: Approximately 19% of the population was immobile with a diffusion coefficient of ∼0.01 μm^2^/sec, whereas the remaining molecules were diffusing with modes at diffusion coefficients of ∼2.5 μm^2^/sec and ∼9 μm^2^/sec (Supplemental Fig. S5C,D; Supplemental Movie S3). In cells treated with Forskolin for 24 h, the fraction of immobile Halo-TFEB molecules increased from 19% to 42% and further raised to 62% in cells treated with Forskolin for 48 h (Supplemental Fig. S5C,D; Supplemental Movies S4, S5). Because our acquired tracking movies included Halo-TFEB trajectories from both the nucleus and cytoplasm, two potential explanations arise from the observed increase in TFEB binding during Forskolin treatment. First, a simple explanation may be that Forskolin-induced relocalization of TFEB causes an increase in the proportion of nuclear trajectories, thus increasing the total bound fraction, whereas the underlying nuclear behavior of TFEB remains the same. It could also be that during Forskolin-induced differentiation, the nuclear population of TFEB also changes its behavior (via post-translational modifications, expression of cofactors, change in the underlying chromatin, etc.), which results in increased binding. To test these two possibilities, we used the collected JF549 images to create conservative, eroded masks of the nucleus in each movie and analyzed only trajectories that were entirely contained within the nuclear mask (Fig. 4C). Interestingly, in the nuclear-masked movies, the nuclear fraction of TFEB also significantly increased its bound fraction in Forskolin-treated relative to DMSO-treated cells, from 37% to 63%, respectively (Fig. 4D,E). Because the DMSO-treated cells contain many fewer trajectories within the nuclear masks than the Forskolin-treated cells, as a control we also reran the saSPT analysis after randomly sampling the same number of trajectories from each data set, limited by the DMSO data set that contained the fewest trajectories (∼20,000) and saw no effect: We found agreement of the bound fraction within 1%, indicating that our statistics are sufficiently powered (Supplemental Fig. S6). These data suggest that Forskolin differentiation induces not only a redistribution of TFEB from the cytosol to the nucleus but also an increase in its propensity for TFEB to associate with chromatin once inside the nucleus.

To determine whether TFEB's increased chromatin association is Forskolin-dependent, we tested TFEB nuclear localization and binding by SMT under other pharmacological perturbations. Consistent with previous reports, we measured increased TFEB nuclear localization upon treatment with either Torin1 or sucrose and Leptomycin B (Supplemental Fig. S7B; Sardiello et al. 2009; Napolitano et al. 2018). In both cases, we also measured an increased association with chromatin of the masked nuclear Halo-TFEB population, indicating that TFEB's increased propensity for chromatin association is not necessarily Forskolin-dependent, but that increased chromatin binding follows from TFEB's relocalization to the nucleus, regardless of chemical stimuli (Supplemental Fig. S7C,D; Supplemental Movies S6, S7). In addition, to investigate TFEB's chromatin binding at endogenous concentrations rather than overexpression, we used CRISPR/Cas9 and AAV-delivered HDR vectors to create endogenously tagged Halo-TFEB knock-in BeWo clones. Despite being regulated by the endogenous promoter, we did notice slightly elevated TFEB protein levels in the Halo knock-in clones compared with unedited cells (1.8-fold to 3.8-fold elevated) (Supplemental Fig. S7E,F). Consistent with our results in the overexpression line, single-molecule tracking of all three Halo-TFEB knock-in clones revealed a significant increase in chromatin-associated nuclear TFEB upon Forskolin treatment (Supplemental Fig. S7G,H). Our results show that during differentiation, increasing nuclear TFEB more readily associates with chromatin in live cells and that this increase can be achieved by drugs that increase TFEB's nuclear concentration, rather than just Forskolin-driven differentiation.

**Figure 5.**
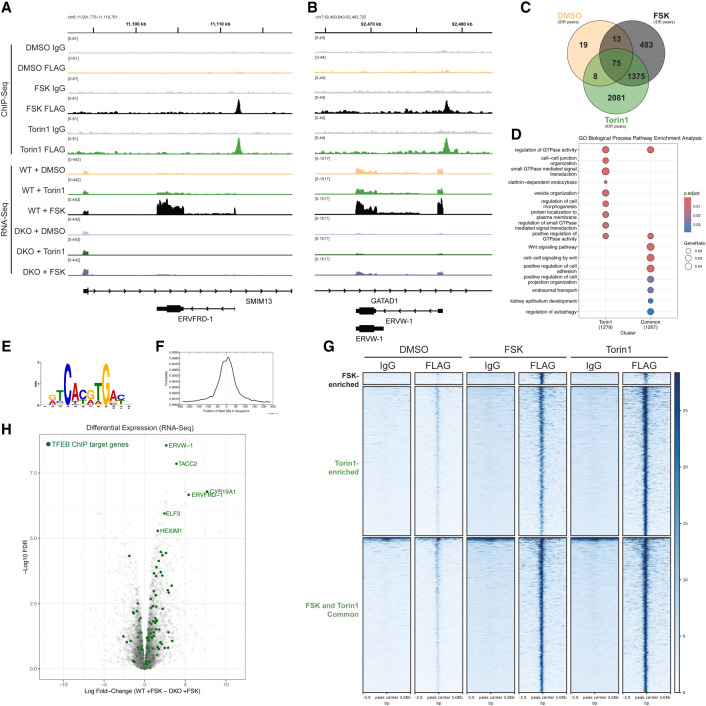
TFEB directly binds the *ERVFRD-1* and *ERVW-1* promoters to regulate their expression. (*A*,*B*) Gene tracks displaying binned reads of 3xFLAG-Halo-TFEB ChIP-seq and RNA-seq in DMSO, 2 h Torin1 treatment, or 48 h Forskolin treatment for the *ERVFRD-1* (*A*) and *ERVW-1* (*B*) loci. Merged replicates 1 and 2 are shown. (*C*) Venn diagram of statistically significant 3xFLAG-Halo-TFEB ChIP-seq peaks, called with IDR analysis (IDR score < 0.05) in each treatment condition. (*D*) GO enrichment analysis of all annotated genes associated with at least one significant 3xFLAG-Halo-TFEB ChIP-seq peak (IDR score < 0.05). Genes with higher Torin1 (2 h) binding or with equivalent Forskolin and Torin1 (common) enrichment (determined by MACS2 bdgdiff) were analyzed separately. The top biological process GO terms are shown. (*E*) The top significant motif from MEME-ChIP analysis of significant 3xFLAG-Halo-TFEB ChIP-seq peak summits (IDR score < 0.05), expanded by 250 bp in both directions and pooled from all treatment conditions. (*F*) Distribution analysis of the top MEME motif “DRTCACGTGAYH” in the analyzed sequences. (*G*) Heat maps of differentially enriched peaks analyzed with MACS2 bdgdiff showing ±3000 bp centered around each peak. Replicates 1 and 2 were merged, and IDR robust peaks (IDR score < 0.05) are shown (see Supplemental Fig. S8 for expanded plots of replicates). (*H*) Volcano plot of WT versus DKO BeWo RNA-seq expression (as shown in Fig. 1A, gray dots) overlaid with annotated TFEB target genes as identified by ChIP (green dots indicate all annotated genes associated with at least one significant 3xFLAG-Halo-TFEB ChIP-seq peak) (IDR score < 0.05). The top regulated TFEB target genes are labeled with their gene name and include *ERVFRD-1* and *ERVW-1*.

Finally, we sought to measure which genomic sites this increasingly chromatin-associated TFEB may be binding to directly during differentiation. We performed ChIP-seq of DMSO-treated, 2 h Torin1-treated, and 48 h Forskolin-treated stably expressing 3xFLAG-Halo-TFEB BeWo cells using a mouse anti-FLAG antibody (Supplemental Fig. S8). In agreement with a strong role of TFEB in stimulating the expression of the two syncytins governing cell–cell fusion, ChIP data show clear TFEB enrichment at the promoter of both *ERVFRD-1* and *ERVW-1* as well as at more distal regions surrounding *MFSD2A* (Supplemental Fig. S9A) upon Forskolin and Torin1 treatments (Fig. 5A,B), suggesting that TFEB is a direct transcriptional regulator of the cell fusion machinery. For subsequent analysis, two biological replicates were merged, and statistically robust peaks were identified with IDR analysis (Supplemental Fig. S8; Li et al. 2011). Of these robust peaks, significant overlap exists between peaks identified in the Forskolin- and Torin1-treated cells, with a larger number of peaks identified as Torin1-specific (Fig. 5C). A de novo MEME-ChIP motif search for TFEB peaks across all conditions confirmed the canonical TFEB motif, an expanded and somewhat flexible E-box motif, centrally enriched around TFEB peak summits (Fig. 5E,F; Machanick and Bailey 2011). Differential TFEB binding after Forskolin and Torin1 treatment was also analyzed using MACS2 bdgdiff, which identified three categories of peaks (Fig. 5G; Gaspar 2018). Differential Forskolin-enriched peaks were few (530); most TFEB peaks were Torin1-enriched (6312) or common to both Torin1 and Forskolin (5343). These differentially enriched peaks were intersected with IDR robust peaks, resulting in 2176 Torin1-enriched peaks and 2242 Forskolin and Torin1 common IDR robust peaks that were mapped to their nearest genomic element for analysis (Supplemental Fig. S9B). Interestingly, some differences emerged in the gene ontology between the mapped genes for Torin1-enriched peaks and the common peaks. Notably, both pathways seemed to converge on GTPase regulation, whereas Torin1-enriched peaks were also enriched in metabolic processes such as endocytosis and vesicle organization, and common peaks pointed to cell adhesion, regulation by Wnt signaling, and autophagy (Fig. 5D). Interestingly, promoter-proximal TFEB peaks were enriched for the more canonical lysosomal and autophagy regulatory GO categories, whereas more distal TFEB peaks were enriched in Wnt signaling, cell adhesion, cell junction assembly, and morphogenesis, suggesting that TFEB's distal regulation may be critical for its role in syncytiotrophoblast differentiation (Supplemental Fig. S9C,D). Overall, we found that in contrast to TFEB's direct binding and regulation of syncytin genes, TFEB's ChIP targets are more broadly uncoupled from TFEB's role in their regulation in BeWo cells (Fig. 5H), suggesting that TFEB can still bind to other gene classes (such as autophagy genes) but is somehow unable to activate them in this cell type.

**Figure 6.**
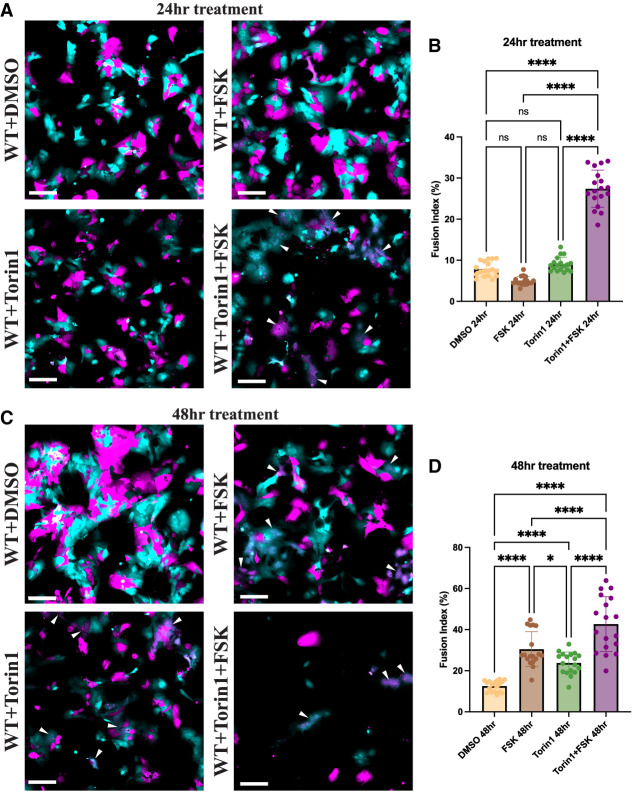
Torin1 treatment increases the rate of syncytiotrophoblast fusion. (*A*) Two-color cell–cell fusion experiments coculturing two populations of mCherry- and GFP-expressing BeWo cells were treated with the indicated compounds for 24 h and then imaged on a spinning-disk confocal microscope. In the merged composite image, the GFP channel is represented in cyan, and the mCherry channel is represented in magenta. White arrowheads indicate fused syncytial areas in the Torin1- and Forskolin-treated wild-type cells. Scale bar, 200 μm. (*B*) Quantification of the two-color cell–cell fusion experiments shown in *A*. (*C*) Same as *A*, except cells were treated with the indicated compounds for 48 h. (*D*) Quantification of the two-color cell–cell fusion experiments shown in *C*. Statistical significance from an ordinary one-way ANOVA with Tukey's multiple comparisons test is shown. (ns) Not significant, (*) *P* < 0.05, (****) *P* < 0.0001.

Finally, because we have shown that 2 h Torin1 treatment already leads to rapid nuclear accumulation of TFEB (Fig. 4B), rapid induction of Syncytin-2 (*ERVFRD-1*) transcript levels (Supplemental Fig. S2B), increased TFEB-chromatin association by fast SMT (Supplemental Fig. S7C), and binding of TFEB at relevant syncytiotrophoblast genes by ChIP (Fig. 5A,B), we hypothesized that the faster relocalization of TFEB in Torin1-treated over Forskolin-treated cells could result in faster rates of cell–cell fusion. Indeed, cotreatment with Torin1 and Forskolin resulted in a synergistic effect at 24 h, with a significantly higher proportion of fused cells than Torin1 or Forskolin treatment alone (Fig. 6A,B). We also found that, consistent with its ability to induce STB marker genes (Supplemental Fig. S2C), 48 h treatment with Torin1 alone resulted in a significant increase in cell fusion, similar to that of Forskolin, at 48 h (Fig. 6C,D). At 48 h, Torin1 and Forskolin together increased fusion even more than either drug alone; however, we noticed that with this high rate of cell fusion, cells began to detach from the dish. The data are consistent with a model that nuclear TFEB is sufficient for cell–cell fusion and that a Forskolin-dependent process synergizes with nuclear TFEB during early time points of cell–cell fusion.

## Discussion

In our present study, we have identified a new direct regulator of the syncytin genes’ (*ERVFRD-1* and *ERVW-1*) expression, encoding for the proteins Syncytin-1 and Syncytin-2 that play an essential role in cell–cell fusion of syncytiotrophoblasts. We demonstrated that TFEB is the predominant TFE family member expressed in BeWo cells and that genetic loss of TFEB causes a significant defect in Syncytin-1 (*ERVW-1*) and Syncytin-2 (*ERVFRD-1*), as well as *MFSD2A* expression upon differentiation, leading to the inability of these cells to fuse (Figs. 1, 2). We further demonstrate in heterologous fusion assays that the Syncytin loss itself is sufficient for the loss in fusion observed in TFEB KO cells (Fig. 2). In producing single TFEB KO clones, our highest knockout efficiency clone was still not 100% complete and showed some residual upregulation of *ERVFRD-1* upon Forskolin treatment (Fig. 1A,H). We postulate that this residual activity in comparison with the complete TFEB/TFE3 DKO clone is due to the small yet detectable residual level of TFEB present rather than the presence of TFE3, which was very lowly expressed at the mRNA level (RPKM < 5) and undetectable by Western blotting (Fig. 1B). Lending strength to this argument are multiple lines of evidence that point to TFEB, rather than TFE3, playing an important role in placental biology. First, reintroduction of TFEB alone in TFEB/3 DKO cells rescued cell–cell fusion (Supplemental Fig. S4). Second, although homozygous TFEB KO in mice is lethal due to placental phenotypes, homozygous TFE3 or MITF KOs show no apparent gestational phenotype (Steingrímsson et al. 2002). Third, a very recent study published during preparation of this article further identified TFEB, and not TFE3, as a genetic hit in a CRISPR screen of genes required for syncytiotrophoblast formation in hTSCs (Shimizu et al. 2023). Finally, in single-cell sequencing of human morula stage embryos, *TFEB* was identified as a trophectoderm (TE)-enriched gene that correlated with the expression of *GATA3*, another trophectoderm marker, whereas *TFE3* did not (Gerri et al. 2020).

Surprisingly, our RNA-seq data identified dysregulated genes that point to a new role for TFEB in controlling expression of key syncytiotrophoblast genes rather than its canonical lysosomal and autophagic gene targets (Fig. 1). Given the seeming importance of lysosomal and autophagy biology in placental cell differentiation, we were surprised by this finding (Oh and Roh 2017; Nakashima et al. 2020b). In our study, even double KO of TFEB and TFE3 did not perturb many lysosomal and autophagy genes or the ability to produce lysosomes during differentiation (Fig. 3). However, ChIP-seq did identify TFEB binding with promoter-proximal enrichment at many lysosomal and autophagic target genes, as previously characterized, as well as binding to many key syncytiotrophoblast genes (Fig. 5). Thus, there seems to be a clear disconnect between TFEB's genetic targets and its transcriptional functions (Fig. 5H). Interestingly, several other recent reports lend credence to the idea of cell type-dependent roles of TFEB outside of its role in CLEAR network regulation. In undifferentiated mouse ESCs, TFEB instead binds to the *Nanog* promoter and controls pluripotency genes, whereas TFE3 rather than TFEB controls the canonical lysosomal and autophagy network genes (Tan et al. 2021). In zebrafish, *tfeb/tfe3a/tfe3b* triple-mutant animals showed almost no changes to steady-state levels of autophagy or lysosomal genes; TFEB dependence of these genes was only observed upon immunological stress (Iyer et al. 2022). Most interestingly, Wnt-driven nuclear localization of TFEB results in an activated subset of Wnt-driven TFEB genes (including genes involved in cell adhesion and secretion) being activated without perturbing expression of lysosomal target genes or lysosomal biogenesis (Kim et al. 2021). These results are consistent with our finding that Torin1- and Forskolin-enriched common genes were enriched in GO categories for Wnt signaling and modulation of cell adhesion (Fig. 5D). Overall, these results lead to several interesting hypotheses for TFEB's altered role in placental cells: It may be that a key TFEB cofactor for regulating the CLEAR network is not present in placental cells, and thus TFEB can bind, but not transactivate, these classical targets. It could also be that placental-specific post-translational modifications alter TFEB's transactivation abilities at specific genes (as is the case for PARsylated Wnt-driven TFEB targets) (Kim et al. 2021). In parallel, it may also be possible that another E-box binding factor (outside of the TFE family) is present in trophoblast cells and instead controls the canonical CLEAR network through direct competition with TFEB at these sites. It could also be that the increase in lysosomal biogenesis during differentiation in syncytiotrophoblasts is controlled post-transcriptionally through stabilization or increased translation of lysosomal proteins. Given the correlated alterations to lysosomal and autophagy pathways noted in some placental diseases, such as preeclampsia, it will be crucial to identify how such changes occur, as our research now suggests that defects in TFEB function would primarily result in defective syncytin expression and cell–cell fusion (also correlated with preeclampsia) but would not be primarily responsible for the lysosomal or autophagy defects seen in some studies (Nakashima et al. 2013, 2020a). These mechanisms will be interesting to distinguish in future studies.

Biophysically, we have shown that nuclear import of TFEB leads to increased chromatin engagement regardless of the chemical initiator of TFEB import (i.e., Forskolin, Torin1, or sucrose and Leptomycin B) (Fig. 4; Supplemental Fig. S7; Fisher et al. 1991). Importantly, we have shown that nuclear localization of TFEB via treatment with Torin1 is sufficient to induce chromatin association and to activate syncytiotrophoblast genes to an extent similar to Forskolin and thus is sufficient to induce cell–cell fusion (Fig. 6; Supplemental Fig. S2). We have shown that rapid inducers of TFEB nuclear localization such as Torin1 can synergize with Forskolin to also increase the rate of cell–cell fusion (Fig. 6). These data suggest that the concentration of nuclear TFEB may be an important rate-limiting step in the induction of cell–cell fusion. Although nuclear TFEB localization with Torin1 treatment alone was sufficient to induce cell–cell fusion, given Torin1's inability to increase cell–cell fusion by 24 h, it is likely that nuclear TFEB is upstream of a second, rate-limiting process that is Forskolin-dependent.

In our study, we have identified TFEB as a novel regulator of human syncytin expression. Because of TFEB's requirement for mouse placentation, we speculate that TFEB could have been independently co-opted to also regulate the phylogenetically unrelated murine syncytins, as several TFEB binding motifs can be found near these genes (Supplemental Fig. S10). Although GCM1 has been shown to be a regulator of both mouse and human syncytins, TFEB's regulation of human syncytins appears orthogonal to existing regulation by GCM1. We found that TFEB depletion did not affect the expression of *GCM1* at the transcript level and that both GCM1 and TFEB bind distinct DNA motifs (Akiyama et al. 1996; Liang et al. 2010). However, ChIP-seq of hTSCs differentiated to STB shows GCM1 occupancy at ∼300 bp upstream of the *ERVFRD-1* promoter, which overlaps with the TFEB peak identified here in our ChIP-seq (Supplemental Fig. S10; Shimizu et al. 2023). Genetically, both individual GCM1 and TFEB KO cells individually are defective in cell–cell fusion, where presumably the other factor is expressed, thus suggesting that both are required for syncytiotrophoblast fusion. It is not yet known whether TFEB may physically interact with GCM1. TFEB protein–protein interaction studies have not yet been performed in trophoblast cells, and because GCM1 is almost exclusively expressed in the placenta (Nait-Oumesmar et al. 2000), any putative interaction has not been documented but will be interesting to investigate.

Finally, therapeutic modalities to prevent or treat pregnancy diseases are desperately needed, and our study may provide a unique therapeutic angle on treating diseases where cell–cell fusion is perturbed, such as preeclampsia and fetal growth restriction. Although correlations have been seen between lysosomal and autophagy defects and placental diseases, research has remained divided on whether and in what cells these effects may be occurring (Oh et al. 2008; Akaishi et al. 2014; Pan et al. 2018; Nakashima et al. 2020a). Drugs that affect TFEB phosphorylation and localization such as Torin1, Rapamycin, and amino acid shortage have been shown to have direct effects on syncytiotrophoblast formation. However, it was previously unclear which factor in the pathway was important for enabling the effects of these pleiotropic drugs (Shao et al. 2021). Upon identifying TFEB as a direct transcriptional effector in trophoblast fusion, this study may reconcile some of these findings by separating TFEB's importance in syncytiotrophoblast differentiation from its role in controlling lysosomal and autophagy genes. Further investigation is warranted because our findings predict that controlling TFEB directly via either genetic perturbations or targeted pharmaceutical interventions may offer a novel approach to regulating cell–cell fusion during syncytiotrophoblast development.

## Materials and methods

### BeWo cell culture

Human placental choriocarcinoma BeWo cells (ATCC CCL-98) were cultured at 37°C and 5% CO_2_ in F-12K medium (Corning or ATCC) supplemented with 10% fetal bovine serum and 10 U/mL penicillin–streptomycin. Cells were passaged by trypsinization every 2–4 days and split at a ratio of 1:3–1:5. Media was changed completely every 24–36 h.

#### Treatments

As indicated in the text, BeWo cells were treated with 20 µM Forskolin dissolved in DMSO (Millipore Sigma F3917), 250 nM Torin1 (Cell Signaling Technology 14379S), or 0.1% DMSO in supplemented F-12K medium. For sucrose treatment, crystalline D-sucrose (Fisher BP220-1) was dissolved in supplemented F-12K medium to 100 mM final concentration, 0.2 µm-filtered, and then added to cells in culture for 24 h total treatment. As indicated, Leptomycin B (InvivoGen inh-lep-10) dissolved in ethanol was used at 20 nM final concentration in 100 mM sucrose-supplemented F-12K medium for 30 min treatment prior to and during imaging.

### Cas9 editing

#### Plasmid method (TFEB/3 DKO #C2)

BeWo cells were plated at 300,000 per well into a 6 well plate and transfected with 10 pU6 sgRNA CBh Cas9 PGK Venus plasmids carrying guides targeting TFEB and TFE3 (Table 1) with Lipofectamine 3000 per the manufacturer's instructions. Two days after transfection, Cas9-Venus^+^ cells (∼6% of the total population) were FACS-sorted into 96 well plates. Cells were grown for ∼2–3 weeks and then genotyped for genomic deletions in TFEB and TFE3 by PCR (Table 2). Clone #C2 was validated by Western blotting and Sanger sequencing (Table 3).

**Table 2.** gPCR primers for TFEB/TFE3 DKO

Primer name	Primer sequence 5′–3′
TFEB_F	AGATGGTGATAAGTGATATGGAGGA
TFEB_R	TTGAATCCTCCCGTTCGCTG
TFE3_F	AGTCGTCATTCACCGAAGGG
TFE3_R	GTTGGTCCCAGGTTAAGGCA

**Table 3.** Sanger sequencing primers for TFEB/TFE3 DKO

Primer name	Primer sequence 5′–3′
TFEB_F_seq	AGCAGAGGGGAAGACAGGAT
TFEB_R_seq	AAGGCACAAAGTGAGGGGTC
TFE3_F_seq	AAAGCGACGCAAACATAGAGG
TFE3_R_seq	CTGTGCTGGGCTGTTCCTAT

#### RNP nucleofection (TFEB KO #c13)

Guide RNAs were ordered from IDT. Cas9 RNP was assembled on ice by adding 0.7 µL of 160 μM crRNA + 0.7 µL of 160 μM trRNA in a PCR strip tube and then incubated in a thermocycler for 30 min at 37°C. Next, 1.4 µL of 40 μM purified Cas9-NLS (Macrolab QB3) was added to the duplexed sgRNA and incubated in a thermocycler for 15 min at 37°C. BeWo cells were split and counted using Trypan blue dead cell exclusion, and 3 × 10^5^ cells per reaction were aliquoted into a 15 mL conical tube and centrifuged at 200*g* for 2.5 min. Cells were resuspended in 20 µL of Lonza SG-supplemented solution per reaction per the manufacturer's instructions. Complexed Cas9 RNP (2.5 µL) was added to 20 µL of BeWo cells in Lonza SG solution, transferred to a Lonza 16 well strip nucleofection cuvette, and nucleofected with a Lonza 4D nucleofector using program CA-137. Immediately following nucleofection, 75 µL of warm F-12K media was gently added to the top of the BeWo cell reaction without mixing, and the cells were allowed to recover for 10 min at 37°C and 5% CO_2_. After recovery, cells were transferred from the cuvette into a 6 well plate for growth.

#### BeWo Halo-TFEB knock-in line generation

BeWo cells were nucleofected with RNP exactly as above to induce cutting near the ATG start site of the TFEB locus. Directly after nucleofection, cells were treated with purified AAV2 vector carrying an HDR template with 500 bp of homology arms specific to the TFEB N terminus flanking an N-terminal 3xFLAG-HaloTag at an MOI of 100,000. After 5 days, cells were stained with 500 nM JF646 HaloTag ligand and FACS-sorted for Halo positivity into single-cell clones. Cells were grown for 2–3 weeks, genotyped using TFEB screening primers (Table 2), and validated by Western blotting and Sanger sequencing.

### Western blotting

Cells were trypsinized, split, and counted, and 1 × 10^6^ cells were centrifuged at 200*g* for 2.5 min, resuspended in 150 µL of PBS + 50 µL of 4× SDS-PAGE loading buffer (200 mM Tris at pH 6.8, 400 mM DTT, 10% B-ME, 8% SDS, 0.4% bromophenol blue, 40% glycerol), boiled on a heat block for 20 min at 100°C, and then centrifuged at 16,000*g* (Eppendorf 5415) for 3 min at 4°C. Supernatant was then transferred to a fresh tube and stored at −20°C. Cell lysate (20 μL) was loaded onto 4%–20% Tris-glycine (Bio-Rad) and transferred onto 0.45 μm nitrocellulose. A solution of 5% bovine serum albumin was used to block membranes for 1 h at room temperature prior to blotting and was used to dilute primary and secondary antibodies. Gels were imaged by addition of Western Lightning ECL reagent on a Chemidoc imaging system.

#### Antibodies

The antibodies used were ACTB (1:5000; Sigma Aldrich A2228), TBP (1:2000; Abcam ab51841), hCGb (1:1000; Abcam ab53087), TFEB (1:1000; Cell Signaling Technology 4240S), TFE3 (1:5000; Abcam ab93808), antirabbit HRP secondary (1:5000; Invitrogen 31462), and antimouse HRP secondary (1:5000; Invitrogen 31430).

### RNA-seq

BeWo cells were plated at 300,000 cells per well into a 6 well plate and allowed to settle 6 h to overnight. Cells were then treated with 0.1% DMSO or 20 μM Forskolin for 48 h. For Torin1 treatment, cells were treated with 0.1% DMSO for 42 h and then switched to 250 nM Torin1 media for 2 h before harvesting. Media for all treatments was changed at 24 h. RNA was extracted from each well using 500 µL of Trizol followed by two sequential chloroform extractions followed by ethanol precipitation per the Trizol user guide (Thermo Fisher MAN0001271). Total RNA was depleted of rRNA using the Illumina rRNA depletion kit (NEB E6310) and then prepared for Illumina sequencing using the NEBNext Ultra II directional RNA library preparation kit for Illumina (E7760) per the manufacturer's protocol. Sequencing was performed by MedGenome on an Illumina NovaSeq with paired-end 150 bp reads. Fastq files were assessed by FASTQC and then pseudoaligned to the hg38 transcriptome using Salmon to quantify transcripts in mapping-based mode (salmon quant -i) using decoy-aware mapping with gencode.v39.transcripts.fa. Differential gene expression analysis was performed with EdgeR in Jupyter Notebook. Statistical comparisons between individual sample conditions were made using EdgeR makeContrasts and then glmQLFit. The reported *P*-values and FDR values were corrected with adjust.method = “BH,” and differentially expressed genes are reported with an adjusted FDR of <0.05.

### qPCR

RNA was harvested from cells using Trizol followed by chloroform extraction and ethanol precipitation. One microgram of RNA from each sample was then reverse-transcribed using iScript (Bio-Rad) following the manufacturer's instructions. Amplification was performed using CFX master mix (Bio-Rad) and a two-step PCR cycling protocol with 60°C annealing. All qPCR primers were evaluated using a dilution series to ensure calculated primer efficiencies of 90%–110%, and amplicons were validated using agarose gel electrophoresis and Sanger sequencing.

### Cell–cell fusion assays

#### Stable cell lines

To create stable cell lines, BeWo cells were grown to ∼50% confluency in a 6 well plate and transduced with 500 µL of crude lentivirus (pHAGE EF1a mCherry IRES Hygro, pHAGE EF1a GFP IRES Hygro, or SFFV GFP1-10 IRES Hygro) in F-12K-supplemented media with 0.8 μg/mL polybrene. After 48 h, cell lines were created by selecting with 200 μg/mL hygromycin for 2 weeks and maintained in hygromycin selection conditions. To create stable HEK293T cells, HEK293T cells were plated to ∼75% confluence in a 6 well plate and transfected with 5 μg of pQCXIP CMV BSR-GFP11 IRES Puro (BSR [blasticidin resistance]) with 7.15 µL of PEIMax in serum-free DMEM. Two days after transfection, cells were selected in 8 μg/mL blasticidin for 2 weeks and maintained in antibiotic selection conditions. The plasmids used are listed in Table 4.

**Table 4.** Plasmids

Reagent	Experiment used	Source
pHAGE L30prom H2B-GDGAGLIN-Halo-V5 IRES Neo	Single-molecule tracking	Tjian-Darzacq laboratory
pHAGE L30prom Halo-NLS IRES Puro	Single-molecule tracking	Tjian-Darzacq laboratory
PiggyBac L30 3xF-Halo-GDGAGLIN-TFEB IRES Puro	Single-molecule tracking	Tjian-Darzacq laboratory, cloned from TFEB plasmid gifted by the Zoncu laboratory
pHAGE EF1a mCherry IRES Hygro	Fusion assays	Tjian-Darzacq laboratory
pHAGE EF1a eGFP IRES Hygro	Fusion assays	Tjian-Darzacq laboratory
pHR-SFFV-GFP1-10 plasmid	Fusion assays	Addgene 80409
pQCXIP-BSR-GFP11	Fusion assays	Addgene 68716
pU6 sgRNA CBh Cas9 PGK Venus	CRISPR editing	Tjian-Darzacq laboratory, derivative of the Zhang laboratory plasmid
pMAX-GFP	Control	Lonza

For two-color fusion experiments, EF1a mCherry IRES Hygro- and EF1a GFP IRES Hygro-expressing BeWo cells were split and mixed at a 1:1 ratio so that 10,000 cells (5000 mCherry + 5000 GFP) were plated per well of a 96 well plate into Perkin Elmer Cell Carrier Ultra plates. For splitGFP fusion, 10,000 SFFV GFP1-10 IRES Hygro BeWo cells were plated into Perkin Elmer Cell Carrier Ultra plates. BeWo cells were allowed to settle and adhere for 3 h to overnight, and media was aspirated and changed to 100 μL/well of F-12K-supplemented media containing 0.1% DMSO (control) or 20 μM Forskolin (treatment). For splitGFP experiments, immediately after changing the media on the GFP1-10 BeWo cells to DMSO or Forskolin, 293T cells stably expressing pQCXIP CMV Blasticidin-GFP11 IRES Puro were split, centrifuged at 200*g* for 2.5 min, and resuspended in a small volume of 293T media (4.5 g/L DMEM containing Glutamax, sodium pyruvate, and PenStrep) and counted using Trypan blue dead cell exclusion. Five-thousand 293T BSR-GFP11 cells in a volume of 1–5 µL were added on top of the BeWo cells. Cells were treated with 0.1% DMSO or 20 μM Forskolin for a total of 48 h before imaging, and media was changed every 24 h. Cells were then imaged on a spinning-disk confocal microscope with a 10× air objective.

#### Confocal imaging

Immediately before imaging, media was changed to 4.5 g/L DMEM without phenol-red containing 10% FBS, 1× Glutamax supplement, and 1× sodium pyruvate supplement plus a final concentration of 2 μg/mL Hoechst 33342 (Invitrogen). High-throughput spinning-disk confocal imaging was performed on a Perkin Elmer Opera Phenix microscope with incubation at 37°C with 5% CO_2_.

For BeWo two-color fusion, a 10× air NA = 0.3 objective was used, and the four channels were acquired (bright-field, EGFP, mCherry, and Hoechst), where Hoechst/bright-field were collected in the same exposure and separated from mCherry/GFP, which were collected in a second exposure. The following illumination settings and filter sets were used: bright-field transmission was imaged with 650–760 nm emission and 100 msec exposure at 20% power; GFP was imaged with 488 nm excitation, 500–550 emission, and 1000 msec exposure at 100% power; mCherry was imaged with 561 nm excitation, 570–630 nm emission, and 1500 msec exposure at 100% power; and Hoechst was imaged with 375 nm excitation, 435–480 nm emission, and 300 msec exposure at 100% power. *Z*-stacks with two to three planes and 7 μm separation were taken to ensure that in-focus images were taken across the plate, and nine central fields of view were acquired per well, which covered almost the entire well area.

For splitGFP cell–cell fusion imaging, a 10× air NA = 0.3 objective was used, and the following laser and filter sets were used to acquire three channels: bright-field transmission was imaged with 650–760 nm emission and 100 msec exposure at 50% power; GFP was imaged with 488 nm excitation, 500–550 emission, and 2000 msec exposure at 100% power; and Hoechst was imaged with 375 nm excitation, 435–480 nm emission, and 400 msec exposure at 100% power. Hoechst/bright-field were collected in the same exposure and separated from GFP, which was collected in a second exposure. *Z*-stacks and fields of view were acquired as above.

#### Image analysis: two-color cell–cell fusion quantification

In the Perkin Elmer Harmony software, maximum projection, bright-field correction, and basic flat-field correction were used for analysis of each field of view. Nuclei were segmented using the “find nuclei” function and method C and selected for area >50 μm^2^ (to eliminate debris). The GFP^+^ and mCh^+^ regions in each image were segmented using an intensity threshold and an area threshold of 100 μm^2^. To avoid artificial regions of mCherry and GFP at nearby cell boundaries, all selected mCherry and GFP regions were resized by eroding the regions by 5 pixels. The pixel-based percentage overlap of selected nuclei with the resized GFP^+^ or mCh^+^ regions was calculated using the “cross population” function. Nuclei were then filtered by using a Boolean operator, and the fusion index was calculated as

$$\mathrm{fusion}\;\mathrm{index}=\frac{++\mathrm{nuclei}\;(>90\text{\%}\;\mathrm{mCh}\;\;\mathrm{overlap}\;\mathrm{AND}\;\;>90\text{\%}\;\mathrm{GFP}\;\mathrm{overlap})}{\mathrm{total}\;\mathrm{fluorescent}\;\mathrm{nuclei}\;(>70\text{\%}\;\mathrm{mCh}\;\;\mathrm{overlap}\;\mathrm{OR}>70\text{\%}\;\mathrm{GFP}\;\mathrm{overlap})}.$$

Each data point was reported as the mean ratio of the fusion index in a given well (averaging across all nine fields of view). For each experiment, six technical replicates were imaged in separate wells, and three biological replicates of each experiment were performed on different days.

#### SplitGFP cell–cell fusion quantification

In the Perkin Elmer Harmony software, maximum projection, bright-field correction, and basic flat-field correction were used for analysis of each field of view. First, the GFP channel image was smoothed with a Gaussian filter (width = 2 pixels). GFP^+^ regions were segmented using an intensity threshold and an area threshold of 100 μm^2^. To account for differences in plating densities, the total DAPI^+^ region was segmented using an intensity threshold, and the area of the thresholded GFP region was normalized to the DAPI area. The reported

$$\mathrm{normalized}\;\mathrm{GFP}\;\mathrm{area}/\mathrm{DAPI}\;\mathrm{area}\;=\;\;\frac{\frac{\mathrm{GFP}\;\mathrm{area}}{\mathrm{total}\;\mathrm{area}}}{\mathrm{normalized}\;\mathrm{DAPI}\;\mathrm{area}},$$

where

$$\mathrm{normalized}\;\mathrm{DAPI}\;\mathrm{area}=\frac{\frac{\mathrm{DAPI}\;\mathrm{area}}{\mathrm{total}\;\mathrm{area}}}{\mathrm{biological}\;\mathrm{replicate}\;\mathrm{mean}\;\left(\frac{\mathrm{DAPI}\;\mathrm{area}}{\mathrm{total}\;\mathrm{area}}\right)}.$$

### Lysosome Imaging

#### Labeling

BeWo cells were plated at 10,000 cells per well into a Perkin Elmer Cell Carrier Ultra 96 well plate. Cells were allowed to adhere 6 h to overnight and then treated with 0.1% DMSO or 20 μM Forskolin for 48 h. Media was changed every 24 h. The media was then replaced with 100 µL/well phenol-free-supplemented DMEM with 1 μg/mL Hoechst 33342 (Invitrogen) and 1:1000 Lysoview-540 (Biotium). Cells were incubated in this medium for 30 min and then imaged without washing.

#### Confocal imaging

For Lysoview-540 imaging, a 40× water NA = 1.1 objective was used and the following laser and filter sets were used to acquire three channels: Lysoview-540 was imaged with 561 nm excitation, 570–630 nm emission, and 500 msec exposure at 100% power; bright-field transmission was imaged with 650–760 nm emission and 300 msec exposure at 100% power; and Hoechst was imaged with 375 nm excitation, 435–480 nm emission, and 200 msec exposure at 70% power. Hoechst/bright-field were collected in the same exposure and separated from GFP, which was collected in a second exposure. Five *Z*-stacks at 0.5 μm separation were acquired and 20 central fields of view were acquired per well, which represents approximately one-fifth of the well.

#### Image analysis: lysosomal spots and area

In the Perkin Elmer Harmony software, individual plane stack processing, bright-field correction, and advanced flat-field correction were used for analysis of each field of view. Because lysosomes could be seen moving during live acquisition of the relatively slow *Z*-stacks, for processing, only one middle plane (plane 2 of five) was used for analysis rather than maximum projections. To count nuclei, the Hoechst channel was Gaussian-smoothed (width = 8 pixels), and nuclei were identified with the “find nuclei” function of method C. Lysosomal spots were identified with the “find spots” function in the Lysoview-540 nm (Cy3) channel using method D. The thresholded lyso region was identified using an absolute intensity threshold. The Lysoview-540 intensity and area were then calculated for each of these regions. The reported value (thresholded lyso region area sum/number of nuclei) was calculated by summing the thresholded lyso region area per field of view and dividing by the total number of nuclei in that field. The reported value (number of lyso spots/number of nuclei per well) was similarly calculated by dividing the total number of spots detected with “find spots” by the total number of nuclei per field of view. Each data point was reported as the mean value across all fields of view in a given well (averaging across all 20 fields of view). For each experiment, six technical replicates were imaged in separate wells, and three biological replicates of each experiment were performed on different days.

#### Image analysis: nuclear area quantification

Using the three channel imaging data acquired for the lysosome imaging, the data set was reprocessed to measure nuclear area in maximum projection *Z*-stacks. In the Perkin Elmer Harmony software, maximum projection, bright-field correction, and advanced flat-field correction were used for analysis of each field of view. Nuclei were segmented using the “find nuclei” function with method C. Nuclei were selected for areas >50 μm^2^ (to eliminate debris), and border objects on the edge of each image were excluded. The area of each nucleus was then calculated. Each data point was reported as the mean nuclear area across all selected nuclei in a given well (averaging across all 20 fields of view). For each experiment, six technical replicates were imaged in separate wells, and three biological replicates of each experiment were performed on different days.

### Nuclear/cytoplasmic Halo-TFEB imaging

#### Stable cell line

Halo-TFEB BeWo cells were created by plating 3E5 wild-type BeWo cells per well into a 6 well plate and transfecting 0.8 μg of PiggyBac L30 promoter 3xF-Halo-TFEB IRES Puro along with 0.4 μg of Super Piggybac transposase expressed on a second plasmid with Lipofectamine 3000 per the manufacturer's instructions (Table 4). Cells were selected in 1 μg/mL puromycin for 2 weeks and maintained in puromycin selection conditions.

#### Labeling

L30 Halo-TFEB BeWo cells were plated at 10,000 cells per well into a Perkin Elmer Cell Carrier Ultra 96 well plate. Cells were allowed to adhere 6 h to overnight and then treated with 0.1% DMSO and 250 nM Torin1 for 1 h, or 20 μM Forskolin for 24 or 48 h. Media was changed every 24 h. The media was then replaced with 100 µL/well supplemented F-12K medium with 500 nM JF646 HaloTag ligand and 1:1000 MembraneSteady-488 nm (Biotium). Cells were incubated in this medium for 30 min, and the medium was changed to phenol-free-supplemented DMEM with 2 μg/mL Hoechst 33342 (Invitrogen) + 1:1000 enhancer (Biotium) and incubated for ∼10–15 min before starting imaging. All media (labeling and washes) contained the indicated drug conditions throughout and during imaging.

#### Confocal imaging

For Halo-JF646 imaging, a 10× water NA = 0.3 objective was used and the following laser and filter sets were used to acquire three channels: JF646 was imaged with 640 nm excitation, 650–760 nm emission for collection, and 1500 msec exposure at 100% power; Hoechst was imaged with 375 nm excitation, 435–480 nm emission, and 400 msec exposure at 100% power; and GFP was imaged with 488 nm excitation, 500–550 emission, and 600 msec exposure at 100% power. GFP/JF646 were collected in the same exposure and separated from Hoechst, which was collected in a second exposure. Three *Z*-stacks at 7.0 μm separation were acquired and nine central fields of view were acquired per well, which covered almost the entire well area.

#### Image analysis: nuclear/cytoplasmic quantification

In the Perkin Elmer Harmony software, maximum projection, bright-field correction, and basic flat-field correction were used for analysis of each field of view. Nuclei were segmented as for nuclear area quantification above. The cytoplasm was then segmented with the “find surrounding region” function with method A. The mean JF646 channel intensity was then calculated within the nuclei and cytoplasm regions. Background was thresholded from all foreground JF646^+^ regions using an intensity cutoff of 200, and the mean JF646 channel intensity was calculated for the background region. The reported ratio was then calculated as

$$\mathrm{background}-\mathrm{corrected}\;\mathrm{nuclear}/\mathrm{cytoplasmic}\;\mathrm{ratio}=\frac{\left(\mathrm{mean}\;\mathrm{nuclear}\;\mathrm{JF}646\;\mathrm{intensity}-\mathrm{mean}\;\mathrm{bkgd}\;\mathrm{JF}646\;\mathrm{intensity}\right)}{\left(\mathrm{mean}\;\mathrm{cytoplasmic}\;\mathrm{JF}646\;\mathrm{intensity}-\mathrm{mean}\;\mathrm{bkgd}\;\mathrm{JF}646\;\mathrm{intensity}\right)}.$$

Each data point was reported as the mean across all selected nuclei in a given well (averaging across all nine fields of view). For each experiment, five technical replicates were imaged in separate wells, and three biological replicates of each experiment were performed on different days.

### Single-molecule tracking and analysis

#### Halo-H2B and Halo-NLS BeWo stable cell lines

Wild-type BeWo cells were transduced with concentrated L30 Halo-H2B IRES Neo or L30 Halo-NLS IRES Puro lentivirus at an MOI of ∼0.5–1.0 (Table 4). Two days after transduction, cells were selected in 1 μg/mL puromycin (Halo-NLS) or 1600 μg/mL neomycin (G418) for 2 weeks and maintained in antibiotic selection conditions.

#### Sample preparation

BeWo cells (200,000) stably expressing L30 Halo-TFEB, Halo-H2B, or Halo-NLS were plated on Matek dishes containing #1.5 coverslip bottoms and allowed to grow overnight. The next day, the media was exchanged for 20 μM Forskolin or 0.1% DMSO-containing supplemented F-12K media, and cells were grown for 48 h. All media was changed every 24 h. For the 24 h Forskolin-treated samples, the sample was treated with 0.1% DMSO for the first 24 h and then changed to 20 μM Forskolin at the 24 h media change time point. Before imaging, cells were stained simultaneously by changing the media to supplemented F-12K media containing 50 nM JF549 and 25 nM (for TFEB) or 5 nM (for H2B and NLS) JFX646 in media containing Forskolin or DMSO, respectively. The slightly higher concentration of JF549 was used to identify nuclei and for nuclear/cytoplasmic segmentation, and JFX646 was used for tracking. Cells were stained with the indicated JF dyes in the incubator for 30 min, rinsed with PBS, and washed in supplemented F-12K media containing 20 μM Forskolin or 0.1% DMSO, respectively, for at least 30 min. Cells were left in this wash media until just before imaging. Just prior to imaging, cells were again rinsed with PBS and then put into phenol-free high-glucose DMEM containing 10% FBS and supplemented with sodium pyruvate and Glutamax with 0.1% DMSO or 20 μM Forskolin, respectively.

#### Acquisition

Single-molecule tracking was performed as previously described by Hansen et al. (2017) with some minor modifications. Single-molecule imaging was performed on a custom-built Nikon TI microscope with a 100×/NA 1.49 oil-immersion TIRF objective (Nikon apochromat CFI apo TIRF 100× oil), an EM-CCD camera (frame transfer mode; vertical shift speed 0.9 μsec; −70°C; Andor iXon Ultra 897), a perfect focusing system to correct for axial drift, and motorized laser illumination (Ti-TIRF, Nikon) to achieve highly inclined and laminated optical sheet illumination. The incubation chamber was maintained at a humidified 37°C atmosphere with 5% CO_2_, and the objective was also heated to 37°C. Excitation was achieved using the following laser lines: 561 nm (1 W; Genesis Coherent) for JF549, 633 nm (1 W; Genesis Coherent) for JFX646, and 405 nm (140 mW; OBIS Coherent) for all experiments. The excitation lasers were modulated by an acousto-optic tunable filter (AA Opto-Electronic AOTFnC-VIS-TN) and triggered with the camera TTL exposure output signal. The laser light was coupled into the microscope by an optical fiber, reflected using a multiband dichroic (405 nm/488 nm/561 nm/633 nm quad-band; Semrock), and focused in the back focal plane of the objective. Fluorescence emission light was filtered using a single bandpass filter placed in front of the camera using the following filters:: Semrock 593/40 nm bandpass filter for JF549 and Semrock 676/37 nm bandpass filter for JFX646. The microscope, cameras, and hardware were controlled through NIS-Elements software (Nikon). The pixel size in this configuration was 160 nm.

For TFEB masking, prior to each movie, a single exposure of ∼20 to 100 msec at 561 nm excitation (10%–20% AOTF) was used to record the JF549 fluorescence signal as cytoplasmic or nuclear within the specified region of interest (ROI) used to subsequently record the SMT movies. Given the variable expression levels between cells, the exposure time and illumination power for this mask exposure were sometimes adjusted manually to ensure the cell intensities were not outside the linear range of the camera. Movies were acquired with stroboscopic illumination and dark-state reactivation of fluorophores during the camera integration time as follows: 1 msec at 633 nm excitation (100% AOTF) of JFX646 was delivered at the beginning of the frame, and 405 nm photoactivation pulses (10% AOTF) were delivered during the camera integration time (∼447 μsec) to achieve a desired mean reactivation of approximately one to 10 molecules per frame. Eight-thousand frames were recorded per cell per experiment. ROIs 150 pixel × 150 pixel were user-selected to be centered on a nucleus (identified via JF549 signal) and were acquired per cell with a camera exposure time of 7 msec. Around 20 cells were recorded for each condition, and each condition was performed in triplicate on separate days for three biological replicates.

#### Localization and tracking analysis with quot

Localization and tracking of molecules were performed using the “quot” package (available at https://github.com/alecheckert/quot). Although tracking was performed over almost the entire movie (starting at frame 100), very conservative tracking parameters were used to ensure that high density at the beginning of the movie did not result in many misconnected trajectories. Specifically, method = “conservative” and max_spots_per_frame = 7 were used for this purpose, which excluded more dense localizations and any ambiguous connections from contributing to valid trajectories. The following configuration settings were specified in the quot config.toml file: [filter] start = 100, method = “identity,” chunk_size = 100; [detect] method = “llr,” *k* = 1.2, *w* = 15, *t* = 18.0; [localize] method = “ls_int_gaussian,” window_size = 9, sigma = 1.0, ridge = 0.001, max_iter = 20, damp = 0.3; [track] method = “conservative,” max_spots_per_frame = 7, pixel_size_um = 0.16, frame_interval = 0.00748, search_radius = 1.0, max_blinks = 0, min_I0 = 0.0, and scale = 7.0.

#### Bayesian inference of Brownian diffusion using saSPT

Modeling of diffusion from trajectories was performed using the Bayesian state array approach encoded in the “saspt” package (available at https://github.com/alecheckert/saspt). To avoid dense localizations or potential misconnected trajectories at the beginning of the movie biasing diffusion states, analysis was excluded for the first 1000 frames of each movie. The StateArray class (from saspt.sa) was used for diffusion analysis, and the following configuration settings were specified: start_frame = 1000, pixel_size_um = 0.16, frame_interval = 0.00748, focal_depth = 0.7, sample_size = 1000000, likelihood = “rbme,” and splitsize = 10. For subsampling the trajectories to the lowest number of any data set, sample_size was set to 20,000 instead. The “bound fraction” was defined as the cumulative posterior occupation for diffusion coefficients <0.1 μ^2^/sec. Movies were collected over three independent days of imaging, with 15–20 cells collected per condition per day.

#### Nuclear masking

Nuclear masks were created in Fiji using the single JF549 exposures collected prior to each movie. For each image, the following processing was performed using an automated batch script in Fiji: run(“Gaussian Blur…,” “sigma = 2”), setAutoThreshold(“Minimum dark”), run(“Convert to Mask”), run(“Fill Holes”), and run(“Options…,” “iterations = 2 count = 1 black pad do = Erode”). Masks were then adjusted by hand as needed. For cells where the TFEB signal was predominantly cytoplasmic, images were inverted before running the process above, or nuclear regions were identified manually.

### 3xFLAG-Halo-TFEB chromatin immunoprecipitation and analysis

#### Cell sample preparation and immunoprecipitation

Stably expressing L30 3xFLAG-Halo-TFEB IRES Puro BeWo cells (passage ∼10) were scaled up to two 15 cm plates per condition. Cells were grown to ∼50% confluence, and the media was changed to 0.1% DMSO or 20 μM Forskolin and treated for 48 h, changing media at 24 h. For Torin1-treated samples, cells were treated with 0.1% DMSO for 46 h and changed to media containing 250 nM Torin1 for 2 h before being fixed. After treatment, media on each 15 cm plate was changed to 20 mL of serum-free F-12K media containing 1% formaldehyde and incubated with shaking for 5 min. Cross-linking was then quenched by adding 10 mL of 0.125 M glycine in PBS and shaking for an additional 5 min. Media was then poured off, and plates were rinsed twice with ice-cold PBS. Finally, 5 mL of PBS plus protease inhibitors (0.25 μM PMSF, 10 μg/mL aprotinin) was added to each plate, and cells were scraped on ice, collected in 15 mL conical tubes, and centrifuged at 1200 rpm for 10 min at 4°C. The supernatant was aspirated, and cell pellets were flash-frozen in liquid nitrogen and stored at −80°C until further processing. Plating, treatment, and fixation of cells were repeated twice for two biological replicates of each condition.

Cell pellets were thawed on ice, resuspended in 2 mL of cell lysis buffer (5 mM PIPES at pH 8.0, 85 mM KCl, 0.5% NP-40; 1 mL/15 cm plate) with protease inhibitors and incubated for 10 min on ice. During incubation, the lysates were pipetted up and down every 5 min. Lysates were then centrifuged at 4000 rpm for 10 min. Nuclear pellets were resuspended in 6 vol of sonication buffer (50 mM Tris-HCl at pH 8.1, 10 mM EDTA, 0.1% SDS) with protease inhibitors, incubated on ice, and sonicated to obtain DNA fragments <1000 bp in length (Covaris S220 sonicator: 20% duty factor, 200 cycles/burst, 150 peak incident power, six to eight cycles of 30 sec on and 30 sec off). Sonicated lysates were cleared by centrifugation, and chromatin (400 μg/antibody) was diluted in RIPA buffer (10 mM Tris-HCl at pH 8.0, 1 mM EDTA, 0.5 mM EGTA, 1% Triton X-1000, 0.1% SDS, 0.1% Na-deoxycholate, 140 mM NaCl) to a final concentration of 0.8 mg/mL and precleared with magnetic Protein G Dynabeads (Thermo Fisher 10009D) for 2 h at 4°C. Precleared lysates were then immunoprecipitated overnight with 4 mg of normal antimouse IgG (Jackson ImmunoResearch 211-032-171) and anti-FLAG [M2] (Sigma Aldrich F1804). About 4% of the precleared chromatin was saved as input. Immunoprecipitated DNA was purified with Qiagen QIAquick PCR purification kit (Qiagen 28106) and eluted in 40 μL of 0.1× TE (1 mM Tris-HCl at pH 8.0, 0.01 mM EDTA). Samples were checked by qPCR together with 2% of the input chromatin and then used in ChIP-seq library preparation.

#### Library preparation

ChIP-seq libraries were prepared independently from two ChIP biological replicates using the NEBNext Ultra II DNA library preparation kit (New England Biolabs E6177L) according to the manufacturer's instructions with a few modifications. As starting material, 20 ng of ChIP input DNA (as measured by NanoDrop) and 20 μL of the immunoprecipitated DNA (spiked with 5 μL of 10 ng/mL sheared *Drosophila melanogaster* DNA) was used. The recommended reagents’ volumes were cut in half. The NEBNext Adaptor for Illumina was diluted 1:10 in Tris/NaCl (pH 8.0; 10 mM Tris-HCl at pH 8.0, 10 mM NaCl), and the ligation step was extended to 30 min. A single purification step with 0.9× vol of Agencourt AMPure XP PCR purification beads (Beckman Coulter A63880) was performed after ligation. DNA was eluted in 22 μL of 10 mM Tris-HCl (pH 8.0), and 20 μL of the eluted DNA was used for the library enrichment step. Library samples were enriched with 11 cycles of PCR amplification in 50 μL of total reaction volume (10 μL of 5× KAPA buffer, 1.5 μL of 10 mM dNTPs, 0.5 μL of 10 mM NEB Universal PCR primer, 0.5 μL of 10 mM index primers, 1 mL of KAPA polymerase, 16.5 μL of nuclease-free water, 20 μL of sample). After amplification, PCR samples were again purified with 0.9× vol of AMPure XP PCR amplification beads and eluted in 33 μL of 10 mM Tris-HCl (pH 8.0). Library concentration was assessed using Qubit dsDNA HS assay kit (Invitrogen Q32851). Libraries were sent to Medgenome, Inc., for fragment analysis, multiplexing, and sequencing on an Illumina NovaSeq 6000 platform (150 bp, paired-end reads). Nine multiplexed libraries (input, mFLAG, IgG in DMSO, and Torin- and Forskolin-treated samples) were pooled and sequenced per lane.

#### ChIP-seq analysis

ChIP-seq raw reads from FLAG-Halo-TFEB BeWo cells treated with DMSO, Torin, and Forskolin were quality-checked with FastQC (version 0.10.1) and aligned to the human genome (hg38 assembly) using Bowtie2 (version 2.3.4.1) with options –local –very-sensitive-local –no-unal –no-mixed –no-discordant -p10 -I 50 -X 1000. Samtools (Li et al. 2009) (version 1.8) was used to create, sort, and index bam files. Peaks were called with MACS2 version 2.1.0.20140616 (-f BAMPE –nomodel) using either the input or IgG DNA as controls. Similar numbers of peaks were called against each control, so the peaks called against the IgG sample were used in further analysis.

Heat maps were created using deepTools (version 2.4.1) (Ramírez et al. 2016). First, bam files were converted to bigWig files with read numbers normalized to 1× sequencing depth, obtaining read coverage per 50 bp bins across the whole genome, using bamCoverage (-of bigwig –binSize 50 –normalizeTo1x 2913022398 –extendReads –ignoreDuplicates). To perform IDR analysis, MACS2 was used to call peaks with a relaxed cutoff (macs2 callpeak -B -p 1e-3 –nomodel -B -p 1e-3 -t). Peaks were then sorted by their *P*-value, and the sorted narrowPeak files were used as inputs for the IDR program. Significant peaks were identified as those with a transformed IDR value of ≥540, which is equivalent to a *P*-value of ≤0.05. For differential enrichment analysis, MACS2 peak calling was repeated with higher stringency but a fixed fragment size (macs2 callpeak -B -t -f BAM -g hs –nomodel –extsize 194). The fixed fragment size was calculated by the average of the predicted fragment length of the FSK FLAG and Torin FLAG sorted bam files (using the command macs2 predictd -i). Differentially enriched peaks were identified by running -g macs2 bdgdiff (options -g 60 -l 120), and the sequencing depths for each condition were identified from the “tags after filtering in control” from the MACS2 callpeak output. Deeptools was used to create heat maps of these differentially enriched peaks using the normalized bamCoverage bigWig files to compute reads across 6 kb centered on TFEB peak and sorted by decreasing TFEB (FLAG) enrichment. Finally, differentially enriched peaks identified by bdgdiff analysis and statistically robust peaks identified by IDR analysis were overlapped using bedtools intersect, and the resulting bed files were mapped to nearby annotated genomic regions using ChIPSeeker with a binding region of −2000 bp to +500 bp using annotated data from TxDb.Hsapiens.UCSC.hg38.knownGene. Enriched GO categories and dot plots were made with ClusterProfiler (Yu et al. 2012, 2015). Summits from these differentially enriched (MACS2 bdgdiff) and statistically robust (IDR *P*-value < 0.05) peaks were derived from MACS2, expanded 250 bp on either side (using the bedtools slop function in Galaxy), filtered for unique entries (using the FASTA “merge files and filter unique sequences” function in Galaxy), and then input into MEME-ChIP (online tool, version 5.5.5) for motif discovery in the HOCOMOCO human (v11 full) database (Machanick and Bailey 2011; Gaspar 2018).

### Statistics and plotting

Western blot images were prepared using Fiji and Adobe Illustrator. RNA-aeq plots were made using ggplot2 in R with Jupyter notebook. Statistical tests and bar charts were made in GraphPad Prism version 10. Genomic track displays were created using IGV viewer (version 2.16.2) and Adobe Illustrator. GO term analysis was performed at https://geneontology.org.

## Supplementary Material

Supplement 1

Supplement 2

Supplement 3

Supplement 4

Supplement 5

Supplement 6

Supplement 7

Supplement 8
