# Supplementary figures and images for: TFEB controls expression of human syncytins during cell–cell fusion
Source: Genes Dev. 2024 Aug 1;38(15-16):718–37. doi: 10.1101/gad.351633.124 (PMC11444194; doi:10.1101/gad.351633.124)

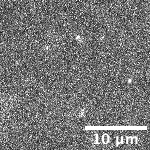

Supplement: Supplement 1 [file Supplemental_Movie1_SMT-example_Halo-NLS.gif]

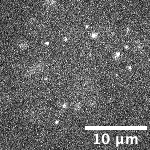

Supplement: Supplement 2 [file Supplemental_Movie2_SMT-example_Halo-H2B.gif]

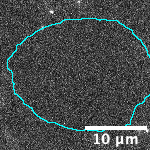

Supplement: Supplement 3 [file Supplemental_Movie3_SMT-example_Halo-TFEB_DMSO_masked.gif]

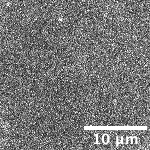

Supplement: Supplement 4 [file Supplemental_Movie4_SMT-example_Halo-TFEB_FSK-24hrs_masked.gif]

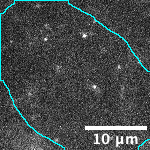

Supplement: Supplement 5 [file Supplemental_Movie5_SMT-example_Halo-TFEB_FSK-48hrs_masked.gif]

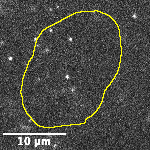

Supplement: Supplement 6 [file Supplemental_Movie6_SMT-example_Halo-TFEB_Torin1_masked.gif]

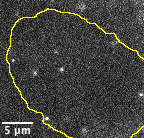

Supplement: Supplement 7 [file Supplemental_Movie7_SMT-example_Halo-TFEB_Sucrose-LMB_masked.gif]
