# Supplementary material for: TFEB controls expression of human syncytins during cell–cell fusion
Source: Genes Dev. 2024 Aug 1;38(15-16):718–37. doi: 10.1101/gad.351633.124 (PMC11444194; doi:10.1101/gad.351633.124)
Supplement: Supplement 8 [file SupplementalFigures_Revision.pdf]

"**TFEB controls expression of human syncytins during cell-cell fusion**" Esbin et al. 2024 - Supplementary Figures

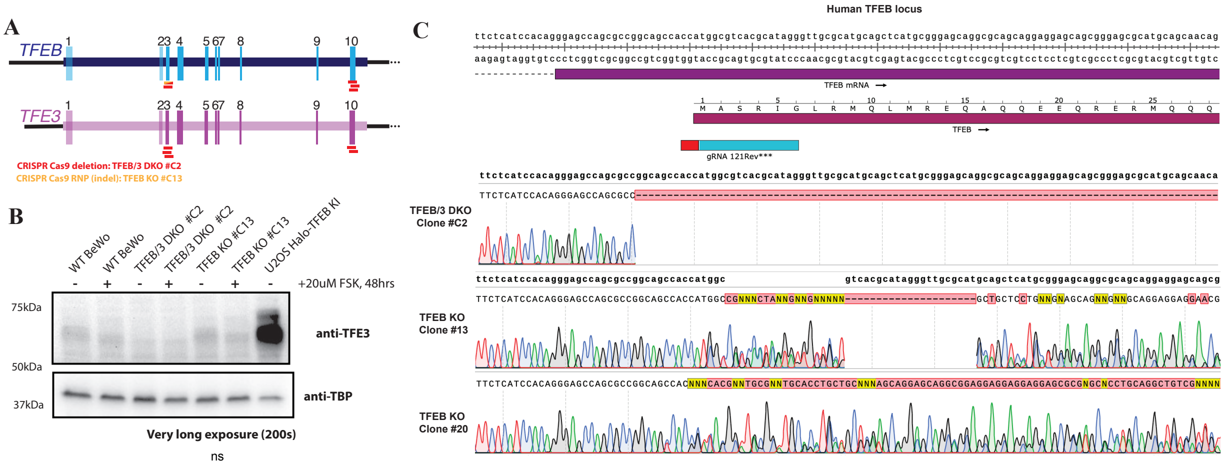

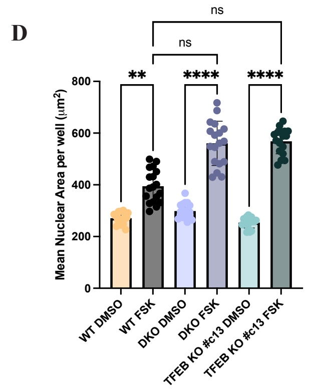

BeWo CRISPR Editing Outcomes

Clone	KO score	CRISPR KO strategy	Allelic outcomes
BeWo TFEB/ TFE3 DKO #C2	100%	Plasmid transfection: Cas9-Venus + 10 U6 sgRNA plasmids	TFEB: 358bp deletion beginning before ATG start in Exon3 through part of Exon 4<br>TFE3: 13kb deletion over entire coding sequence (homozygous KO)
BeWo TFEB KO #c13	95%	Cas9 RNP Nucleofection with 1 sgRNA	Predicted by Synthego ICE:<br>42% alleles +1 insertion; 18% alleles -2 insertion<br>30% alleles -2 insertion; 5% alleles -2 insertion
BeWo TFEB KO #c20	95%	Cas9 RNP Nucleofection with 1 sgRNA	Predicted by Synthego ICE:<br>44% alleles -5 insertion; 10% alleles -11 insertion<br>20% alleles -11 insertion; 2% alleles -5 insertion<br>17% alleles -11 insertion; 2% alleles -5 insertion

Figure S1. Further assessment of TFEB/TFE3 perturbation in BeWo cells. A) Diagram depicting the two strategies and sgRNAs and their positions relative to TFEB and TFE3 used for CRISPR-mediated gene silencing in BeWos. **B)** Long exposure of the same TFE3 western blotting in Figure 1B in wild-type and CRISPR KO BeWo cells, and in U2-OS cells. **C)** Sanger sequencing traces of TFEB/TFE3 DKO and TFEB KO clones showing indels caused by CRISPR Cas9 within the TFEB coding locus and a table describing the generation method and editing outcomes predicted by Synthego ICE analysis for the BeWo clones used throughout this publication. **D)** Quantification of high-throughput confocal imaging measuring the mean per-well nuclear area for wild-type, DKO, and TFEB KO BeWo cells treated with DMSO or Forskolin for 48hrs. Statistical significance from Kruskal-Wallis ANOVA test with Dunn's multiple comparison test is shown where ns = not significant, * = p<0.05, ** = p<0.01, *** = p<0.001, **** and p<0.0001.

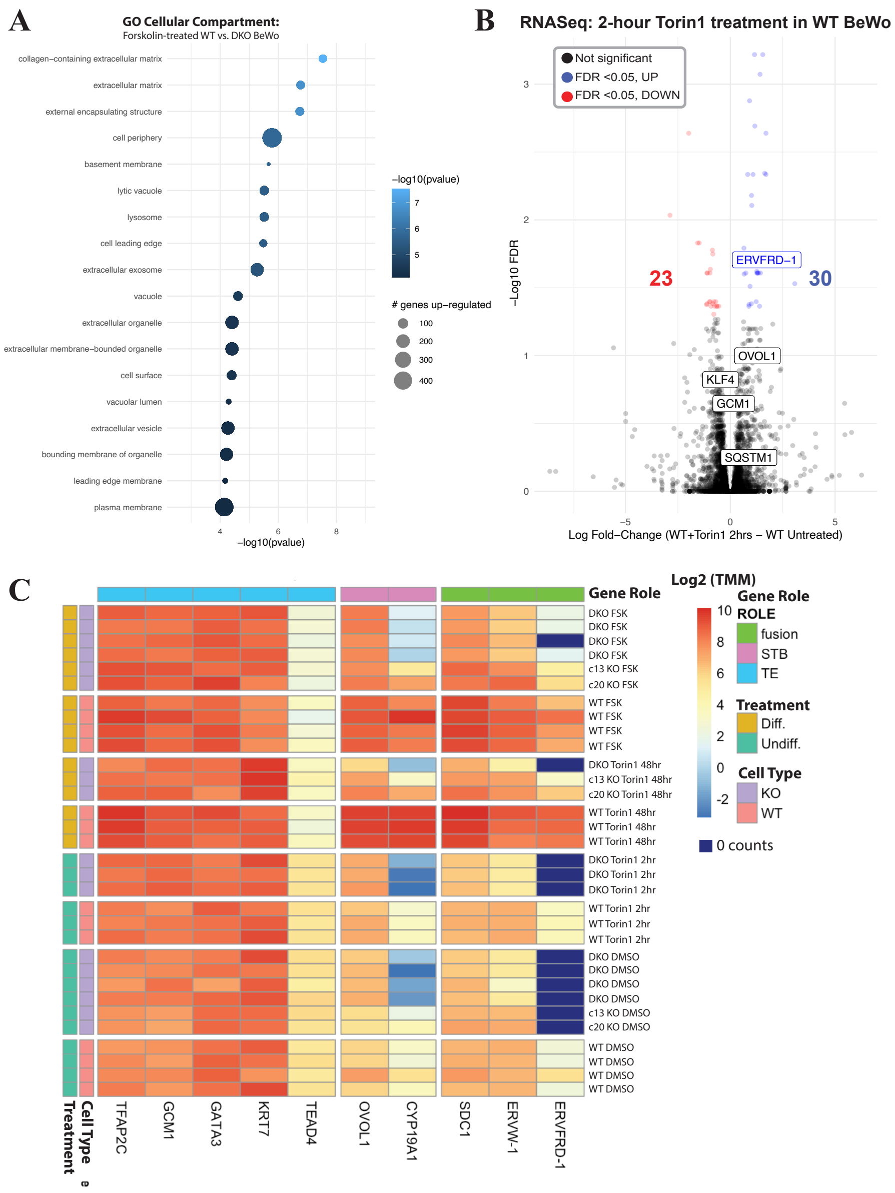

Figure S2. Further assessment of RNASeq in TFEB/TFE3 DKO BeWo cells. A Top 10 enriched GO Terms for Cellular Compartment of genes which are upregulated in the wild-type Forskolin-treated BeWos compared to the Forskolin-treated TFEB/TFE3 DKO cells.) **B)** Volcano plot of RNA-Seq data comparing 2-hour Torin1-treated wild-type BeWo cells with 2-hour Torin1-treated TFEB/TFE3 DKO BeWo cells. Genes significantly upregulated in the Torin1-treated DKO cells are shown in red, and genes significantly downregulated in the Torin1-treated DKO cells are shown in blue. **C)** Heatmap of gene expression of syncytiotrophoblast marker (STB), fusion-related genes, or trophectoderm-expressed genes (TE) (Log2 TMM values from RNA-Seq) in wild-type and TFEB KO or TFEB/3 DKO BeWo cells in untreated, Torin1-treated (2-hours or 48-hours) or differentiated conditions.

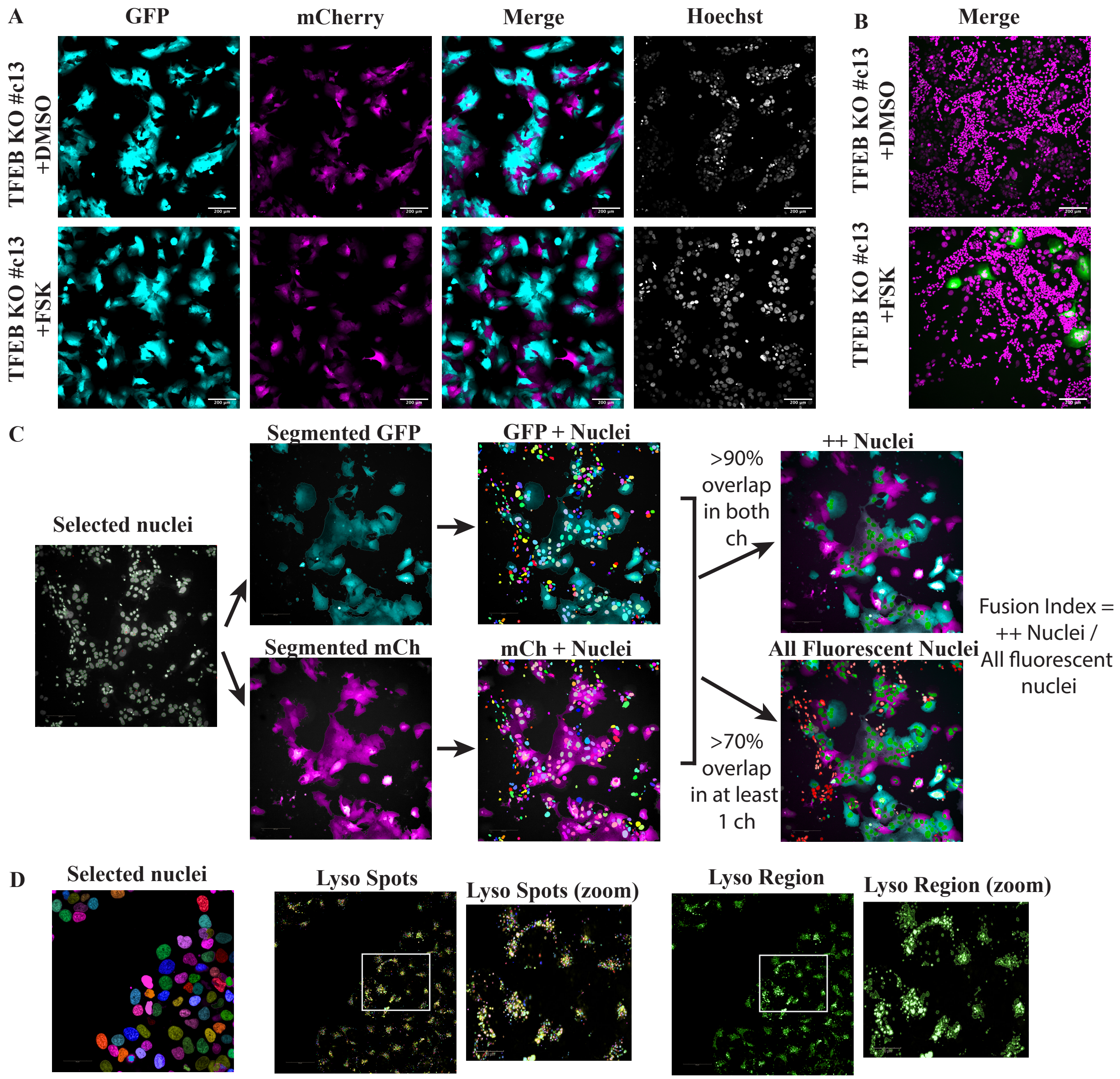

Figure S3. TFEB KO cell-cell fusion defects and segmentation analysis pipelines. A) Two color cell-cell fusion experiments co-culturing two populations of mCherry- and GFP-expressing TFEB KO #c13 BeWo cells were imaged on a spinning disk confocal microscope. Individual channels are shown in grayscale. In the merged composite image, the GFP channel is represented in cyan and the mCherry channel is represented in magenta. White arrows indicate fused syncytial areas in the Forskolin-treated wild-type cells. Scale bar = 200µm. **B)** Split-GFP cell-cell fusion experiments co-culturing GFP1-10 expressing TFEB KO #c13 BeWo cells with GFP11-expressing 293T cells were imaged on a spinning disk confocal microscope. In the merged composite image, the GFP channel is represented in green and the Hoechst channel is represented in magenta. Fused syncytial areas are shown by the reconstitution of GFP fluorescence shown in green. Scale bar = 200µm. Quantification of B and C are shown in Fig. 3C,D. **C)** Overview and example of the two color cell-cell fusion segmentation and quantification pipeline. Selected nuclei are shown in green outline. Subsequently, GFP (cyan) and mCherry (magenta) channels are separately thresholded and filtered to yield single channel-positive regions. Nuclei (rainbow) are overlaid on each channel and those with >90% overlap in both channel regions are deemed ++ (top right, green nuclei) while those with >70% overlap in either channel are

defined as fluorescent nuclei (bottom right, green nuclei; excluded nuclei which do not meet this criteria are shown in red). In panel C only, images were adjusted for easy visualization of segmentation and gamma = 2.0 applied. **D)** Example of segmentation of live lysosomal imaging. Selected nuclei (left, rainbow) are used segmented and the counts used for normalization of cell density. Lysoview-540 imaged in the Cy3 channel (yellow) are segmented with a spot detector (rainbow). A close-up view of the segmentation quality is shown (yellow, right). To quantify the total area occupied by lysosomes, the Lysoview-540-positive region is thresholded and filtered (green) and the area summed. A close-up view of the segmentation quality is shown (green, far right).

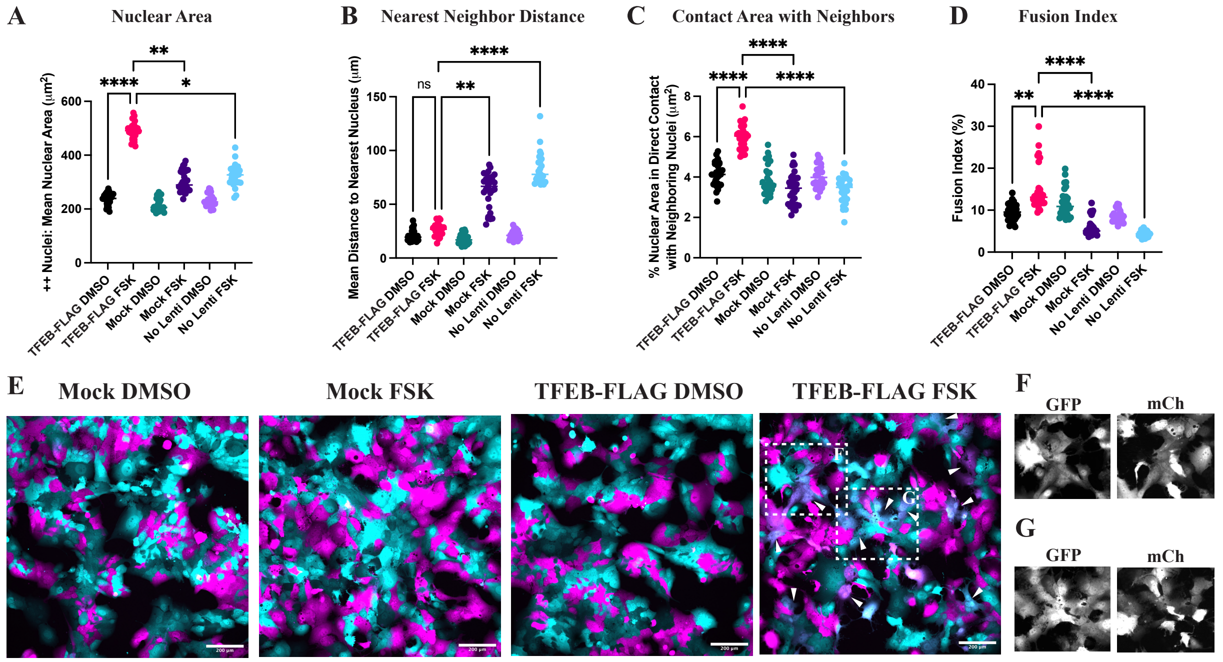

Figure S4. Re-expression of TFEB rescues syncytiotrophoblast phenotypes in DKO BeWo cells . A) Quantification of cellular and nuclear phenotypes that occur during syncytiotrophoblast formation including the enlargement and clustering of nuclei were imaged in DKO #C2 BeWo cells treated with TFEB-FLAG lentivirus, an inert lentivirus (encoding for GFP11, “Mock”), or no lentivirus. Quantified phenotypes shown include Nuclear Area (**A**), the nearest-neighbor distance between nuclei (**B**), the clustering of nuclei by their contact with nearby nuclei (**C**), and cell-cell fusion (**D**). **E)** Two color cell-cell fusion experiments of mCherry- and GFP-expressing DKO #C2 BeWo cells transduced with an inert lentivirus (encoding for GFP11, “Mock”) or TFEB-FLAG were imaged on a spinning disk confocal microscope. Individual channels are shown in grayscale. In the merged composite image, the GFP channel is represented in cyan and the mCherry channel is represented in magenta. White arrows indicate fused syncytial areas. Scale bar = 200µm. **F-G)** Zoomed-in panels shown individual mCherry and GFP channels in grayscale side-by-side to show the overlap in fused two-color-positive regions.

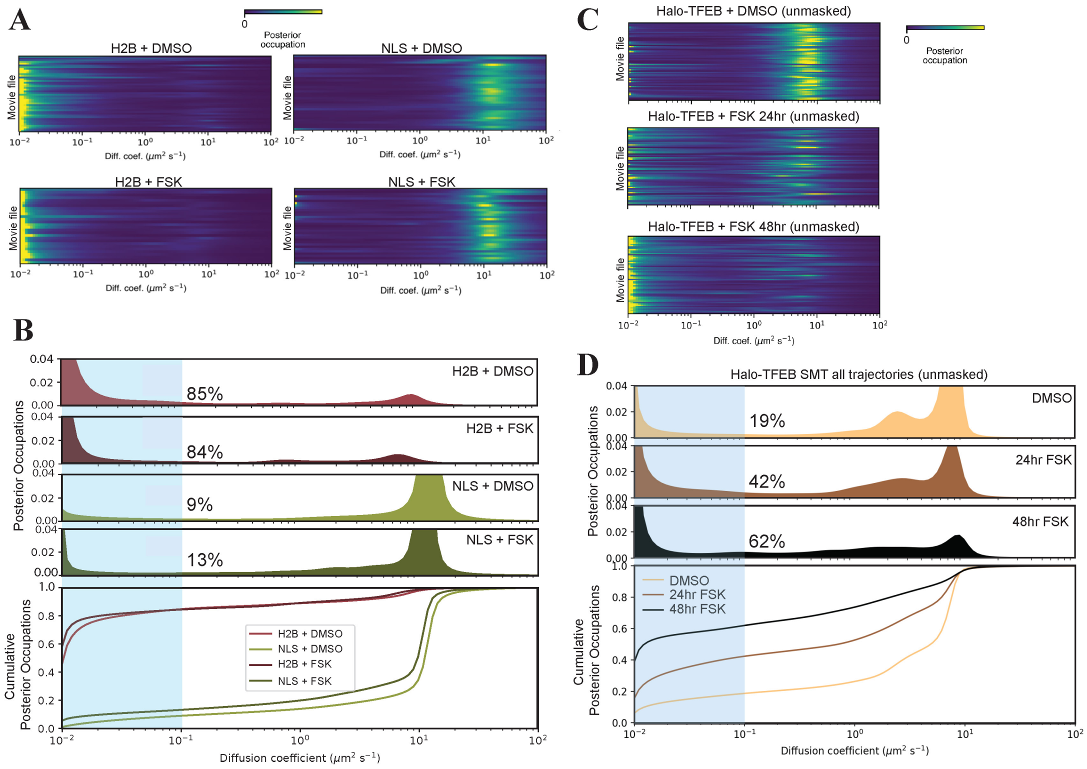

Figure S5. Extended single molecule imaging data: controls and unmasked trajectories. A) Heatmaps of diffusion coefficients following Bayesian analysis of single molecule trajectory data of control BeWo cells expressing Halo-H2B or Halo-NLS treated with DMSO or Forskolin for 48hrs. Each row corresponds to the distribution of posterior occupations for a single movie file. **B)** The distribution of diffusion coefficient occupancies for trajectories of Halo-NLS and Halo-H2B treated with DMSO or Forskolin for 48hrs. The fraction bound (calculated by the fraction of the distribution with a diffusion coefficient $<0.1\mu m^2/s$) is shown highlighted with the blue region and the quantified values displayed as black percentages. The cumulative distribution function (CDF) of this same distribution is shown below. **C)** Heatmaps of diffusion coefficients following Bayesian analysis of all unmasked single molecule trajectory data of Halo-TFEB in BeWo cells treated with different drug conditions. Each row corresponds to the distribution of posterior occupations for a single movie file. **D)** The distribution of diffusion coefficient occupancies for all unmasked trajectories of Halo-TFEB treated with DMSO or Forskolin for 48hrs. The fraction bound (calculated by the fraction of the distribution with a diffusion coefficient $<0.1\mu m^2/s$) is shown highlighted with the blue region and the quantified values displayed as black percentages. The cumulative distribution function (CDF) of this same distribution is shown below.

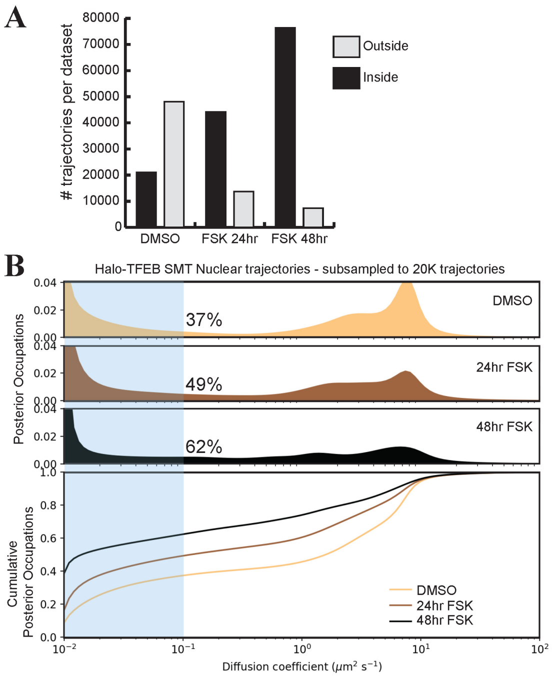

Figure S6. Subsampling nuclear Halo-TFEB trajectories to assess impact of fewer nuclear trajectories on Bayesian modeling. A) A barchart of the summed total number of trajectories in each dataset across replicates and cells after masking segmentation. Trajectories fully encompassed inside the nuclear mask (and thus included in Fig 4E) are shown in black bars. **B)** The distribution of diffusion coefficient occupancies after re-analysis of only 20,000 randomly selected masked nuclear trajectories of Halo-TFEB treated in each condition. The fraction bound (calculated by the fraction of the distribution with a diffusion coefficient $<0.1 \mu m^2/s$) is shown highlighted with the blue region and the quantified values displayed as black percentages. The cumulative distribution function (CDF) of this same distribution is shown below.

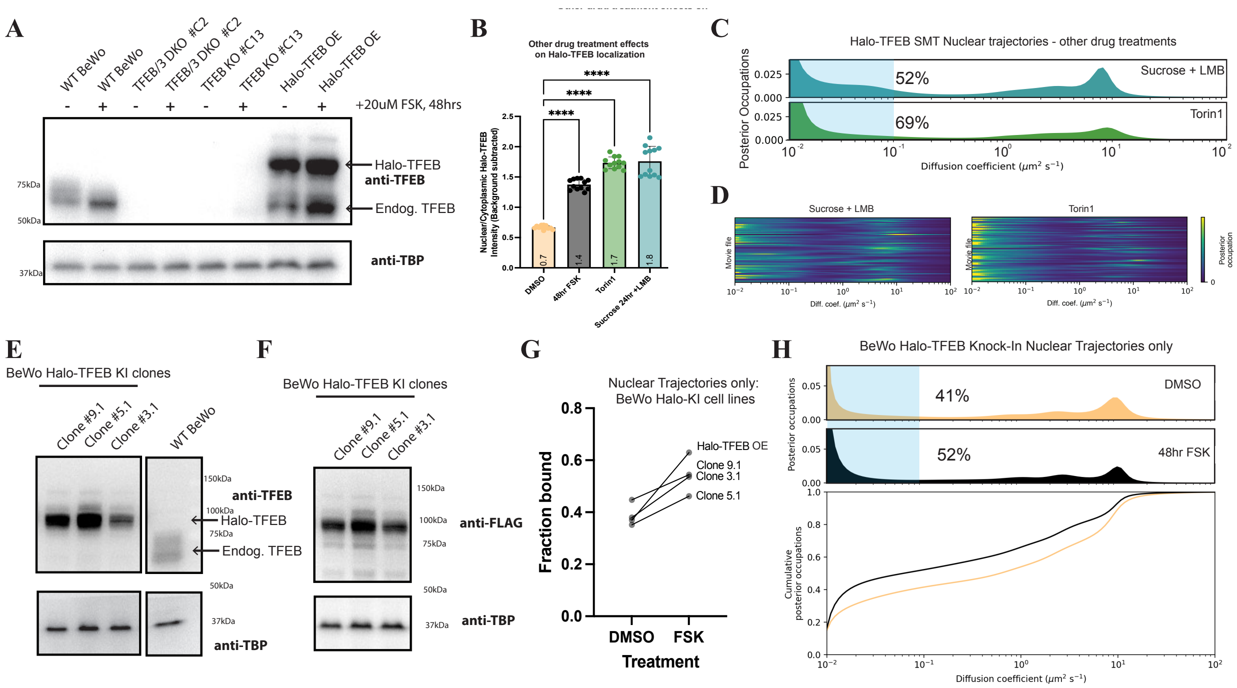

Figure S7. Nuclear TFEB drives TFEB chromatin association upon other drug conditions and in Halo knock-in cells. A) Western blot of stable overexpressing BeWo 3xFlag-Halo-TFEB cell line compared to wild-type and knockout BeWo cell lines. **B)** Quantification of live cell spinning disk confocal imaging of Halo-TFEB after treatment with specified drug treatment. Statistical significance from an ordinary one-way ANOVA with Tukey's multiple comparisons test is shown where ns = not significant, * = $p<0.05$, ** = $p<0.01$, *** = $p<0.001$ and **** = $p<0.0001$. **C)** The distribution of diffusion coefficient occupancies for nuclear segmented trajectories of Halo-TFEB in other drug conditions. The fraction bound (calculated by the fraction of the distribution with a diffusion coefficient $<0.1\mu m^2/s$) is shown highlighted with the blue region and the quantified values displayed as black percentages. **D)** Heatmaps of diffusion coefficients following Bayesian analysis of single molecule trajectory data of Halo-TFEB in BeWo cells in alternative drug conditions. Each row corresponds to the distribution of posterior occupations for a single movie file. **E-F)** Western blot of endogenously tagged 3xFlag-Halo-TFEB BeWo clones **G)** Fraction of TFEB molecules bound in nuclear-segmented single molecule tracking movies of stable overexpressed and endogenous knock-in BeWo Halo-TFEB cell lines. **H)** The distribution of diffusion coefficient occupancies for nuclear segmented trajectories of Halo-TFEB pooled across three knock-in clones. The fraction bound (calculated by the fraction of the distribution with a diffusion coefficient $<0.1\mu m^2/s$) is shown highlighted with the blue region and the quantified values displayed as black percentages.

A

Replicate	Sample	Overall alignment rate
Rep 1	DMSO mFlag	76.07%
Rep 1	FSK mFLAG	74.98%
Rep 1	Torin1 mFLAG	75.95%
Rep 1	DMSO IgG	89.92%
Rep 1	FSK IgG	64.55%
Rep 1	Torin1 IgG	68.79%
Rep 1	DMSO Input	94.72%
Rep 1	FSK Input	94.73%
Rep 1	Torin1 Input	94.36%
Rep 2	DMSO mFlag	74.56%
Rep 2	FSK mFLAG	61.96%
Rep 2	Torin1 mFLAG	67.57%
Rep 2	DMSO IgG	68.00%
Rep 2	FSK IgG	69.06%
Rep 2	Torin1 IgG	70.17%
Rep 2	DMSO Input	77.71%
Rep 2	FSK Input	79.01%
Rep 2	Torin1 Input	76.03%

B

Condition	Called against (control sample)	# IDR significant peaks called (p-value<0.05)
DMSO	IgG	116
FSK	IgG	1948
Torin1	IgG	3539
ALL	IgG	5603
DMSO	Input	197
FSK	Input	1656
Torin1	Input	3393
ALL	Input	5246

C

Condition	# Peaks differentially enriched (MACS2 bdgdiff)	# diff-enriched peaks called as IDR-significant peaks (p<0.05)	# diff-enriched & IDR-significant peaks mapped to promoters (TSS +/- 2000bp)	# diff-enriched & IDR-significant peaks mapped to distal regions (TSS >2000bp)
FSK-enriched	530	175	18	155
Torin1-enriched	6312	2175	444	1662
FSK and Torin1 common	5343	2212	454	1684

D

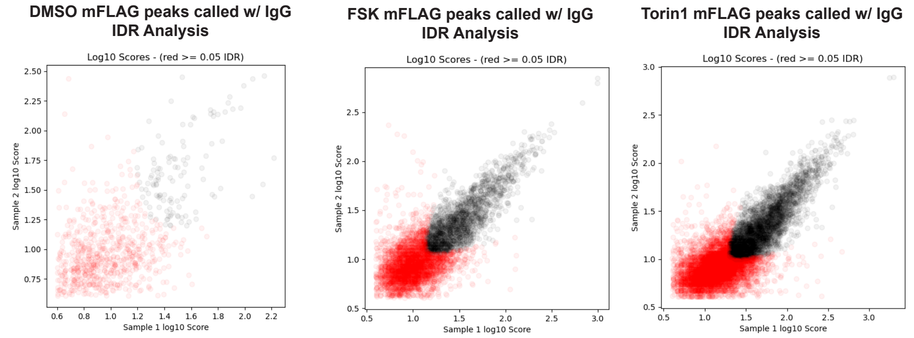

E

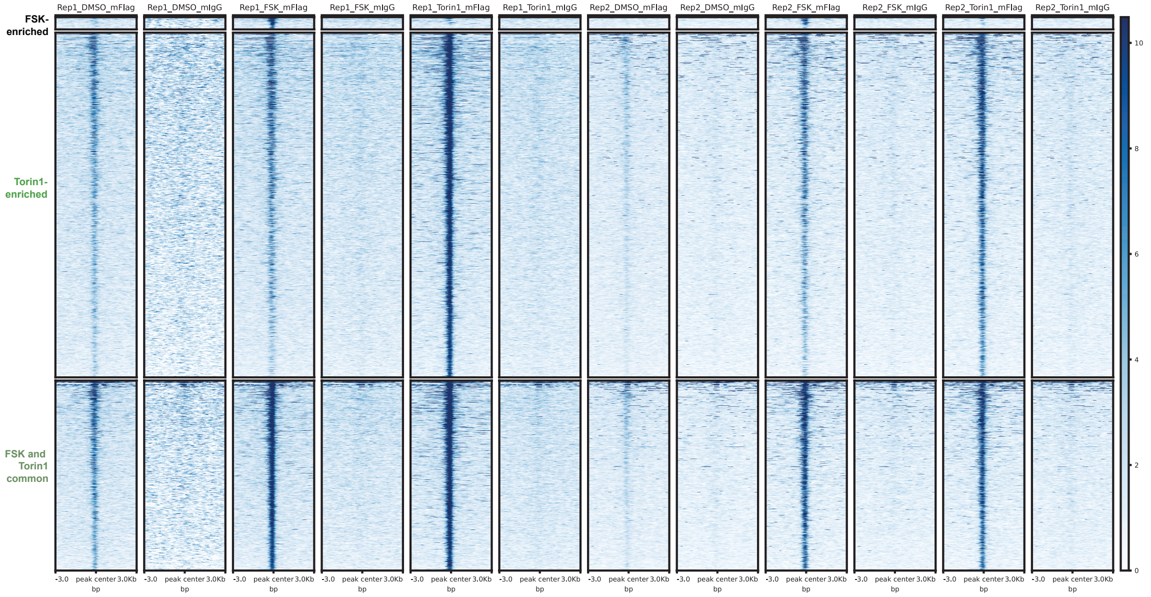

Fig S8. 3xFlag-Halo-TFEB ChIP quality control and peak filtering. A-C) Tables indicating the sequencing depths and subsequent filtering steps in ChIP-Seq processing and analysis. **D)** IDR Analysis of robust peaks across both ChIP-Seq replicates. Sample1 and Sample2 correspond to each replicate and black points indicate robust peaks with an IDR score of <0.05. **E)** Heatmap of differentially enriched peaks analyzed with MACS2 bdgdiff showing +/- 3000bp centered around each peak for all replicates independently.

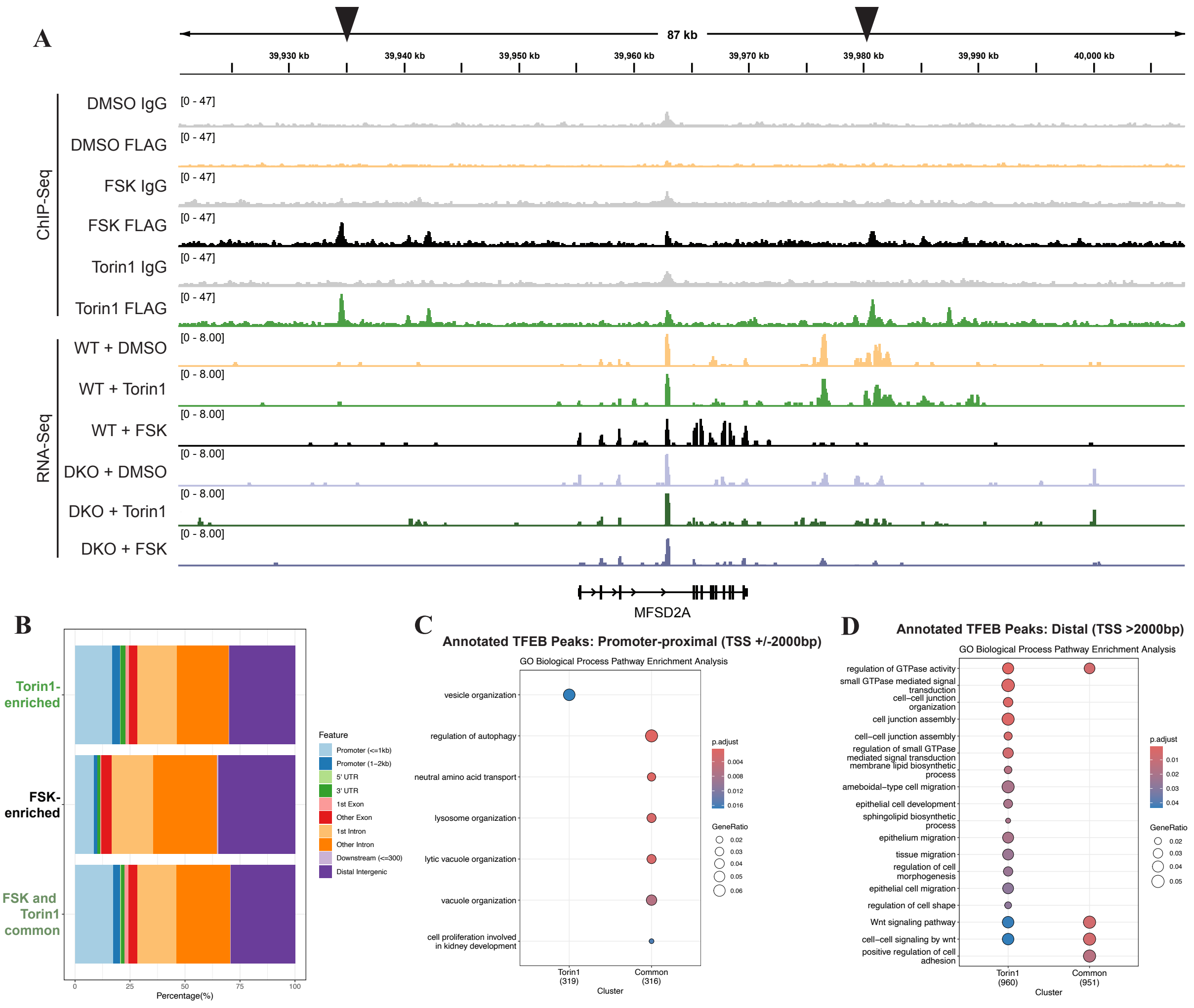

Fig S9. 3xFlag-Halo-TFEB ChIP gene targets, including MFSD2A.

A) Gene tracks displaying binned reads of 3xFlag-Halo-TFEB ChIP-Seq and RNA-Seq at the MFSD2A locus. Merged Replicates 1 and 2 are shown. Putative TFEB-binding peaks are indicated with a black triangle. **B)** Annotation of IDR robust (score <0.05) peaks to genomic regions using ChIPSeeker. **C)** GO enrichment analysis of all IDR robust (score <0.05) TFEB peaks annotated to promoters (TSS +/- 2000bp) across all samples (Forskolin, Torin1, and DMSO treatments). **D)** GO enrichment analysis of all IDR robust (score <0.05) TFEB peaks not annotated to promoters (distal regions = TSS> 2000bp) across all samples (Forskolin, Torin1, and DMSO treatments).

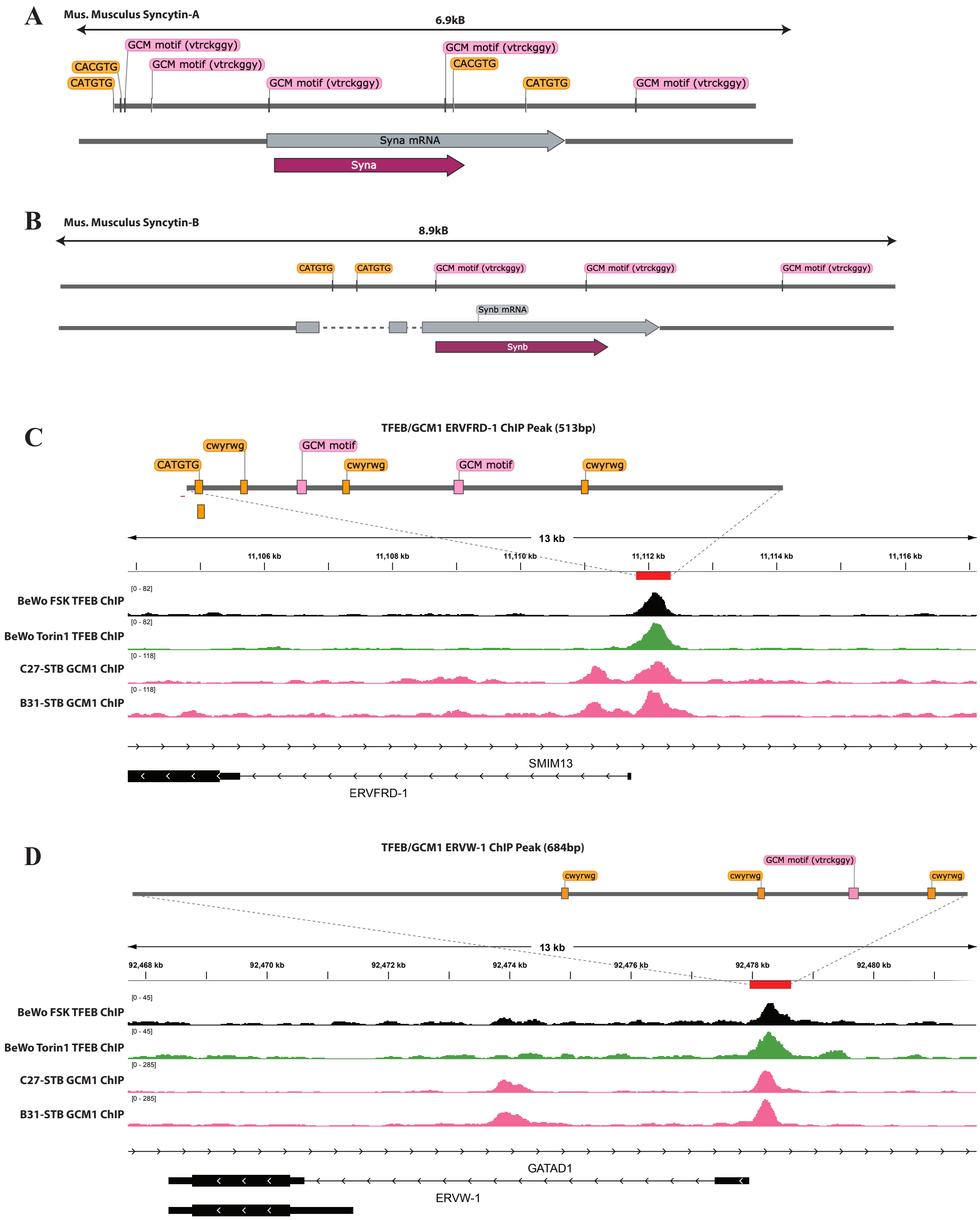

Fig S10. Position of TFEB-binding motifs within human and mouse syncytin genes. A-B) Position of potential TFEB-binding motifs (E-box and expanded E-box motifs in orange) and potential GCM1-binding motifs (GCM motif sequences in pink) proximal to the murine Syncytin genes SynA (A) and SynB (B). **C-D)** TFEB BeWo ChIP data generated in this study compared

to GCM1 ChIP in human trophoblast stem cells (Shimizu, et al. 2023) showing overlapping regions of TFEB and GCM1 binding in the Syncytin-2 (C) and Syncytin-1 (D) promoters with potential annotated binding sites based on sequence motifs.

Supplemental Movies:

Supplemental_Movie1_SMT-example_Halo-NLS.gif
Single molecule tracking of Halo-NLS in stably expressing BeWo cells stained with JF646 and acquired with stroboscopic illumination at 7ms exposures. Scale bar is 10µm and movie is displayed at 50 frames per second.

Supplemental_Movie2_SMT-example_Halo-H2B.gif
Single molecule tracking of Halo-H2B in stably expressing BeWo cells stained with JF646 and acquired with stroboscopic illumination at 7ms exposures. Scale bar is 10µm and movie is displayed at 50 frames per second.

Supplemental_Movie3_SMT-example_Halo-TFEB_DMSO_masked.gif
Single molecule tracking of Halo-TFEB in stably expressing BeWo cells stained with JF646 and acquired with stroboscopic illumination at 7ms exposures. Scale bar is 10µm and movie is displayed at 50 frames per second. The nuclear mask used for selecting nuclear-only trajectories in subsequent analyses is displayed as an outline overlayed on the image.

Supplemental_Movie4_SMT-example_Halo-TFEB_FSK-24hrs_masked.gif
Single molecule tracking of Halo-TFEB in stably expressing BeWo cells treated with 20µM Forskolin for 24 hours stained with JF646 and acquired with stroboscopic illumination at 7ms exposures. Scale bar is 10µm and movie is displayed at 50 frames per second.

Supplemental_Movie5_SMT-example_Halo-TFEB_FSK-48hrs_masked.gif
Single molecule tracking of Halo-TFEB in stably expressing BeWo cells treated with 20µM Forskolin for 48 hours stained with JF646 and acquired with stroboscopic illumination at 7ms exposures. Scale bar is 10µm and movie is displayed at 50 frames per second. The nuclear mask used for selecting nuclear-only trajectories in subsequent analyses is displayed as an outline overlayed on the image.

Supplemental_Movie6_SMT-example_Halo-TFEB_Torin1_masked.gif
Single molecule tracking of Halo-TFEB in stably expressing BeWo cells treated with 250nM Torin1 for 1 hour stained with JF646 and acquired with stroboscopic illumination at 7ms exposures. Scale bar is 10µm and movie is displayed at 50 frames per second. The nuclear mask used for selecting nuclear-only trajectories in subsequent analyses is displayed as an outline overlayed on the image.

Supplemental_Movie7_SMT-example_Halo-TFEB_Sucrose-LMB_masked.gif
Single molecule tracking of Halo-TFEB in stably expressing BeWo cells treated with 100mM sucrose-supplemented media for 24 hours, then sucrose-supplemented media plus 20nM Leptomycin B for 30 minutes stained with JF646 and acquired with stroboscopic illumination at 7ms exposures. Scale bar is 10µm and movie is displayed at 50 frames per second. The nuclear mask used for selecting nuclear-only trajectories in subsequent analyses is displayed as an outline overlayed on the image.
